# VISTA immune checkpoint blunts radiotherapy-induced antitumor immune response

**DOI:** 10.1016/j.celrep.2025.115893

**Published:** 2025-06-27

**Authors:** Dhanya K. Nambiar, Sainiteesh Maddineni, Jimpi Langthasa, Hongbin Cao, Vignesh Viswanathan, Junyan Liu, Md Tauhidul Islam, Nishant Mehta, Jessica Frank, Alexander Real, Tia Cheunkarndee, Eyiwunmi Eghonghon Laseinde, Bhushan Dharmadhikari, Dipti Thakkar, Jerome D. Boyd-Kirkup, Andrey Finegersh, Vasu Divi, John B. Sunwoo, John Aleman, Xiao-Jing Wang, Christina Kong, Lei Xing, Jennifer R. Cochran, Quynh-Thu Le

**Affiliations:** 1 Department of Radiation Oncology, Stanford University School of Medicine, Stanford, CA, USA; 2 Department of Otolaryngology, Head and Neck Surgery, Stanford University School of Medicine, Stanford, CA, USA; 3 Department of Bioengineering, Stanford University Schools of Engineering and Medicine, Stanford, CA, USA; 4 Hummingbird Bioscience, Singapore, Singapore; 5 Department of Pathology, University of Colorado, Anschutz Medical Campus, Aurora, CO, USA; 6 Department of Pathology and Laboratory Medicine, University of California, Davis, School of Medicine, Davis, CA, USA; 7 VA Northern California Health Care System, Sacramento, CA, USA; 8 Department of Pathology, Stanford University School of Medicine, Stanford, CA, USA; 9 Lead contact

## Abstract

Radiotherapy (RT) is a key treatment for solid neoplasms like head and neck cancer (HNC), but it can also activate and recruit immunosuppressive myeloid cells, causing treatment failure. In this study, we examine the role of V-domain immunoglobulin suppressor of T cell activation (VISTA) on myeloid cells during RT. We discovered high VISTA expression on myeloid cells in the tumor microenvironment (TME) of both murine and human HNC, with RT increasing VISTA^+^ myeloid cells in the TME and circulation. Compared to VISTA^+/+^ mice, VISTA^−/−^ mice showed improved tumor control with RT, with their macrophages and neutrophils exhibiting antitumorigenic properties on sc-RNA-seq analysis, especially with RT. Combining anti-VISTA antibodies (active or silent Fc) with RT (fractionated or ablative) significantly decreased tumor volume compared to either treatment alone in multiple preclinical models (HNC, breast cancer, and colorectal cancer), enhancing systemic antitumor immune response with augmented intra-tumoral T cell function through myeloid repolarization. Targeting VISTA could improve the efficacy of RT.

## In brief

Nambiar et al. showed that radiotherapy induces VISTA^+^ immunosuppressive myeloid cells, limiting antitumor immunity. Genetic deletion or antibody-mediated blockade of VISTA synergizes with radiotherapy to enhance T cell responses and myeloid repolarization, improving tumor control across models and supporting VISTA as a promising combination partner for radiotherapy treatment regimen.

## Graphical Abstract

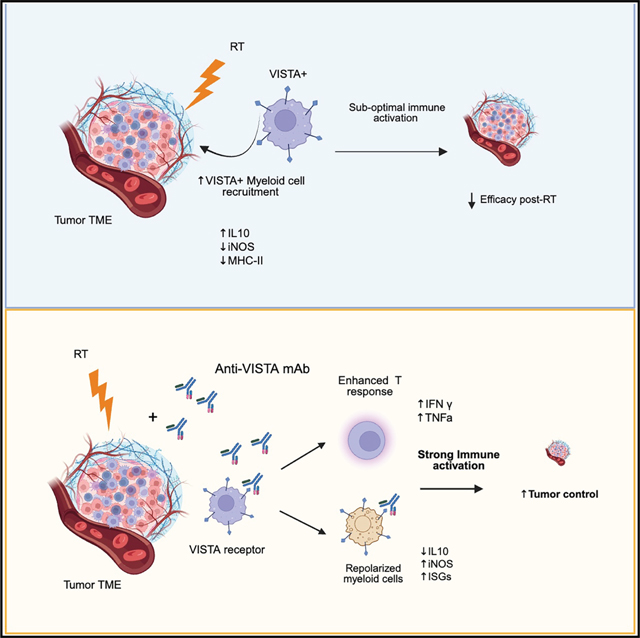

## INTRODUCTION

Radiotherapy (RT) is a cornerstone for solid tumors, including head and neck cancer (HNC), but improving its effectiveness remains challenging. In addition to killing cells, RT can alter immune responses by activating both pro-inflammatory and immunosuppressive pathways. Combining RT with immunotherapy aims to enhance antitumor immune responses while minimizing immunosuppression. While preclinical studies show strong synergy between RT and immune checkpoint inhibitors (ICIs), such as antiprogrammed cell death protein 1 (PD1) or anti-PD ligand 1, most clinical trials have been largely negative,^1–3^ with some exceptions.^4^

A key challenge is that RT, despite inducing immunogenic cell death and releasing tumor antigens, paradoxically recruits myeloid-derived suppressor cells (MDSCs) to the tumor microenvironment (TME), which promotes immunosuppression and tumor progression.^5^ MDSCs are now recognized as major modulators of RT response, and their accumulation after RT can hinder treatment outcomes. Targeting immune checkpoints on myeloid cells, such as VISTA (V-domain immunoglobulin [Ig] suppressor of T cell activation), could reverse immunosuppression, restore T cell function, and improve RT efficacy.

VISTA (or PD-1H or B7-H5) is an immunosuppressive molecule found on various hematopoietic cells, especially in the myeloid lineage.^6–9^ It acts as a potent immunosuppressive checkpoint, inhibiting T cell activation and facilitating tumor immune evasion. VISTA blockade could restore T cell function and enhance antitumor immunity.^8,10,11^ Anti-VISTA antibodies have demonstrated promising results in augmenting immunotherapy and improving response to chemotherapy.^8,12–14^ However, its specific role in RT response, particularly regarding MDSCs and tumor-associated macrophages (TAMs), remains under investigation.

Utilizing mouse models and patient samples of HNC, we show that RT increases VISTA expression in myeloid cells, resulting in more VISTA^+^ myeloid cells in the TME and in the blood. VISTA deletion or blockade significantly improves the tumor control and survival outcomes of RT. Single-cell RNA sequencing (scRNA-seq) analyses highlight that VISTA blockade alters the immunosuppressive functions of MDSCs and TAMs, showing intricate interactions between VISTA on myeloid cells and their role in RT response. The study lays the ground-work for innovative strategies of targeting VISTA-mediated immunosuppression to improve the efficacy of RT.

## RESULTS

### VISTA is a highly expressed immune checkpoint on myeloid cells in TME

**Figure 1.**
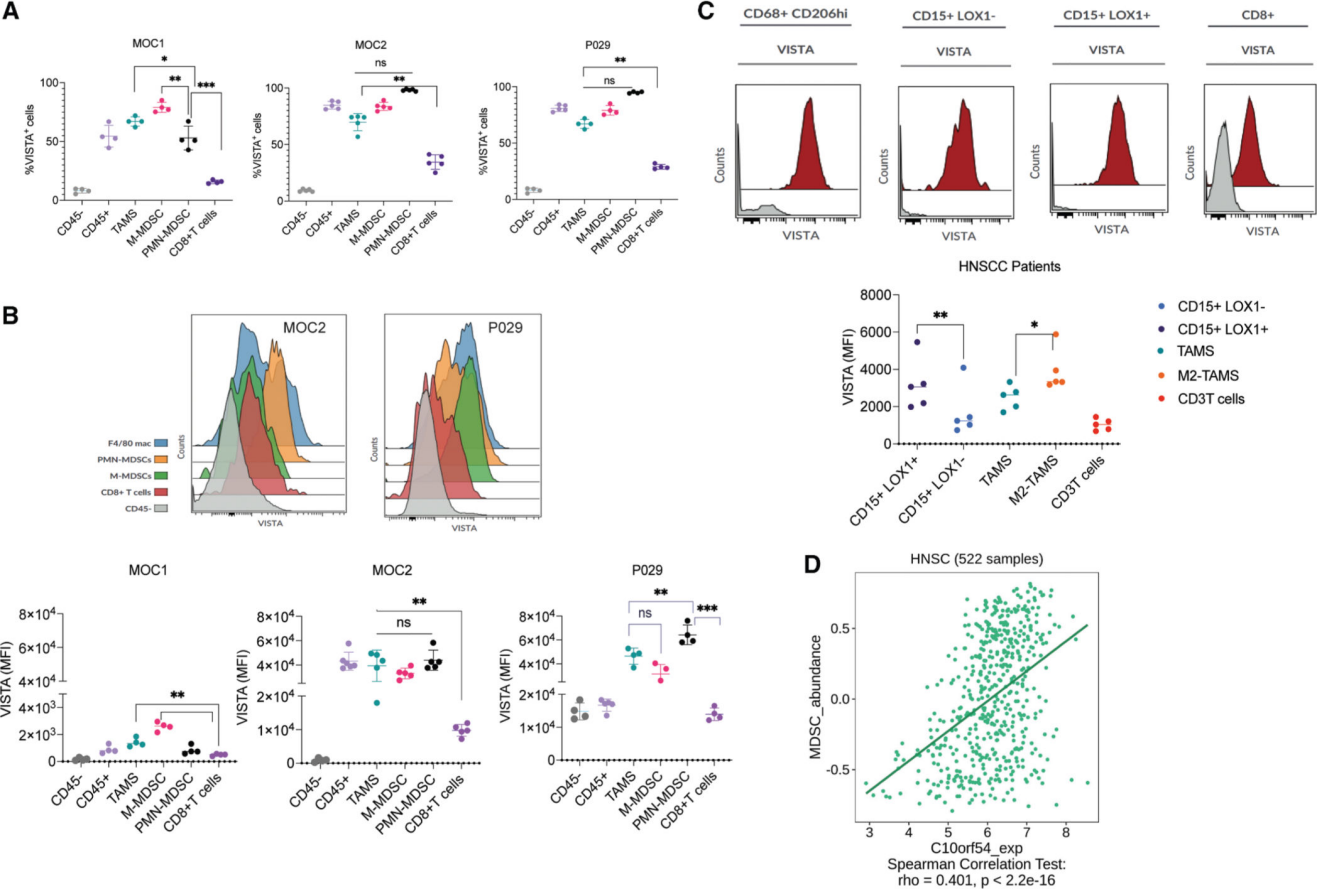
VISTA is a highly expressed immune checkpoint on myeloid cells in the head and neck cancer microenvironment (A) Quantification of VISTA^+^ cells in different immune cell subsets from multiple head and neck cancer (HNC) mouse tumor models (*n* = 4–5). (B) Quantification of mean fluorescence intensity (MFI) of VISTA expression on the different immune cell types in the tumor microenvironment of MOC1, MOC2, and P029 HNC mouse tumor models (*n* = 4–5). (C) Quantification of VISTA expression on CD15^+^LOX1^+^ (PMN-MDSCs), CD15^+^LOX1^−^ (neutrophils), CD8^+^ macrophages (TAMs), CD68^+^CD206^+^ (M2 macrophages or TAMs), and T cells in the tumors of patients with head and neck squamous cell carcinoma (HNSCC) (*n* = 5). *p < 0.05; **p < 0.01. (D) Correlation of VISTA (C100rf54) gene expression with MDSC abundance in The Cancer Genome Atlas dataset of HNSCC using TIMER2.0 analyses. All data were presented as mean ± SD. *p < 0.05; **p < 0.01; ***p < 0.001. All experiments were repeated at least two times, and representative results are shown.

HNCs have significant infiltration of myeloid cells.^15^ To investigate VISTA expression, three murine HNSCC models, MOC1, MOC2, and P029, representing diverse TMEs, were used. MOC1 is immunologically ‘‘hot,’’ with high CD8^+^ T cells and major histocompatibility complex (MHC) class I, while MOC2 and P029, with their regulatory T cell (Treg) infiltration, lower MHC class I expression, and myeloid enriched, represent a more immunosuppressive, or ‘‘cold,’’ TME.^16^ These models represent the diverse human papillomavirus-negative (HPV^−^) head and neck squamous cell carcinoma (HNSCC) with heterogeneity in mutational signature. Flow cytometry showed that VISTA expression was confined mostly to the immune cells (CD45^+^), with minimal presence on cancer or stromal (CD45^−^) cells. Among the immune cells, ∼60%–100% of myeloid cells, including TAMs, polymorphonuclear MDSCs (PMN-MDSC), and monocytic MDSCs (M-MDSC) were VISTA^+^ (Figure 1A). We also examined intra-tumoral CD8^+^ T cells, as some studies indicated potential VISTA expression^17,18^; however, the fraction of these cells expressing VISTA was significantly low (∼10%–30%; Figure 1A) compared to myeloid cells. Notably, aggressive/metastatic models (MOC2, P029) had higher percentages of VISTA^+^ myeloid cells compared to the indolent MOC1, although direct comparisons were limited by differing tumor growth rates. Mean fluorescence intensity (MFI) analysis showed that VISTA expression was highest in MDSCs, followed by TAMs and T cells (Figure 1B).

We extended these findings to human HNSCC samples (patient demographics in Table S1), where high VISTA levels were observed in CD15^+^LOX1^+^ cells (PMN-MDSCs) and TAMs. Interestingly, VISTA MFI was higher in CD15^+^LOX1^+^ cells (PMN-MDSC) compared to CD15^+^LOX1^−^ (neutrophils). Similarly, VISTA expression was higher in M2 TAMs compared to the total TAM population, suggesting a relationship between VISTA expression and suppressive function (Figures 1C and S1). Analysis of the HNSCC-The Cancer Genome Atlas dataset confirmed a direct correlation between VISTA gene (*VSIR*) expression and MDSC abundance, reinforcing that VISTA is a prominent negative immune checkpoint on tumor-associated myeloid cells in HNSCC (Figure 1D). These results highlight VISTA’s central role in immune suppression within the TME, particularly via myeloid cells.

### RT enhances VISTA^+^ myeloid cells in TME

**Figure 2.**
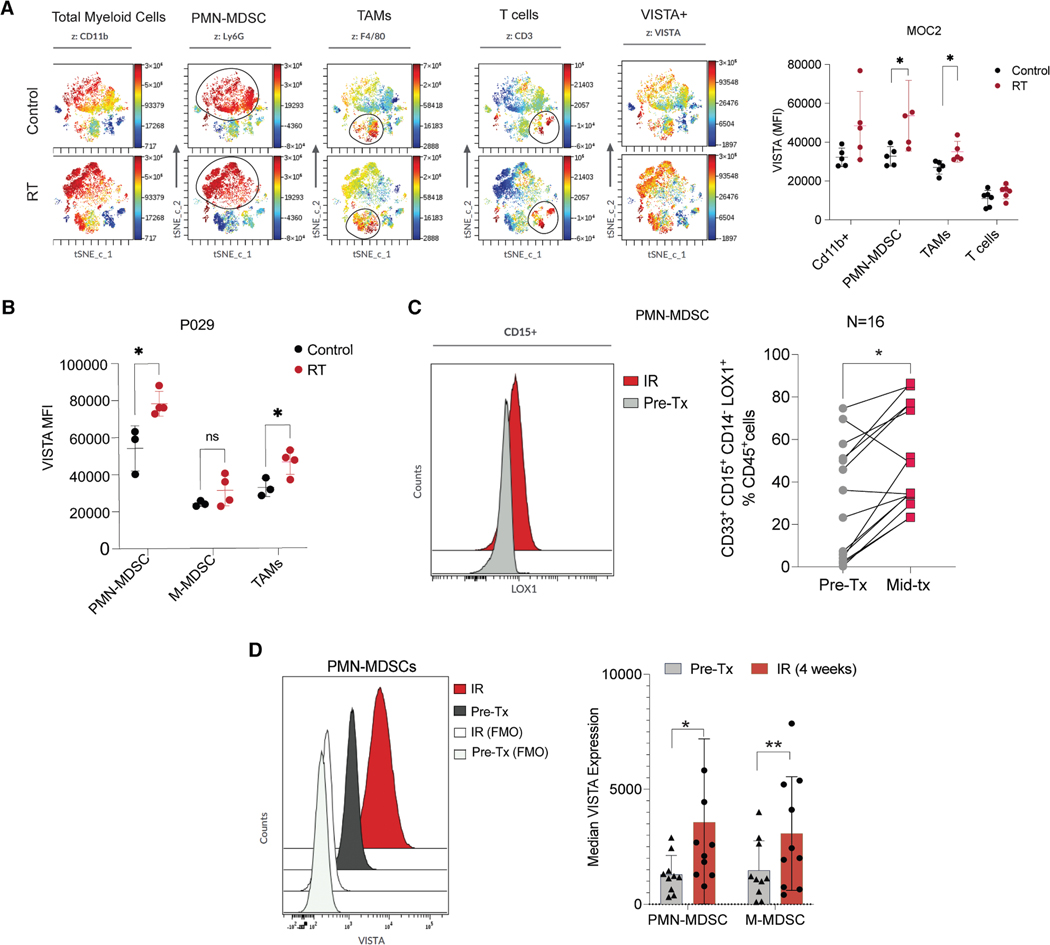
Radiation increases VISTA expression on myeloid cells (A) Quantification of VISTA expression (MFI) in tumor-infiltrated immune cells 2 weeks post-treatment with either sham radiation (top) or 3Gyx5 radiation (bottom) in MOC2 mouse tumor model. *n* = 4–6. (B) Quantification of VISTA expression on myeloid cells 2 weeks post-RT treatment in P029 model. *n* = 3–4. (C) Quantification of PMN-MDSCs (CD33^+^CD15^+^CD14^−^LOX1^+^) levels in the blood of patients with HNSCC at pre-treatment vs. mid-RT treatment. (D) Quantification of VISTA expression levels (MFI) on PMN-MDSCs (CD33^+^CD15^+^CD14^−^LOX1^+^) and M-MDSCs in the blood of patients with HNSCC at pre-treatment vs. mid-RT treatment (4 weeks into the RT course). *p < 0.05; **p < 0.01. All animal experiments were repeated at least two times, and representative results are shown.

Studies have shown that RT can induce immunosuppression by recruiting MDSCs to the TME.^19^ MDSCs also display significant phenotypic plasticity based on factors in the TME such as pH, hypoxia, and inflammatory mediators.^20^ To understand how RT affects VISTA expression on cells in the TME, we irradiated MOC2 tumors with fractionated RT (3Gyx5) when they reached ∼100 mm^3^ and then evaluated them by flow cytometry at ∼2 weeks after the start of RT. The number of MDSCs (primarily PMN-MDSCs) in the TME rose after RT treatment. t-Distributed stochastic neighbor embedding analyses showed that VISTA expression was increased significantly in PMN-MDSCs and TAMS (Figure 2A). Similar effects were observed in the P029 HNSCC model, suggesting that RT increases VISTA expression (Figure 2B). To verify whether this occurs in patients, we evaluated blood samples collected before and during the RT course (∼3–4 weeks into the course of RT ± chemotherapy) from 16 patients with HNSCC, whose demographics are shown in Table S2. The circulating levels of CD33^+^CD15^+^CD14^−^LOX1^+^ cells (PMN-MDSCs) rose in most patients from pre- to mid-treatment time points (Figure 2C). Analysis of VISTA expression in these samples showed significantly increased VISTA expression (MFI) in both circulating PMN- and M-MDSCs (CD33^+^CD15^−^CD14^+^HLA-DR^−^) during RT treatment (Figure 2D).

### VISTA^−/−^ mice show enhanced tumor control post-RT treatment

To evaluate the role of VISTA in RT response, we tested fractionated (3Gyx5) and single-dose (15 Gy) RT in MOC2 tumors (C57BL6 wild-type [WT] mice), observing comparable VISTA induction (Figure S2A). Given its aggressive phenotype, myeloid-rich immunosuppressive microenvironment (high MDSCs, Tregs, low MHC class I/CD8^+^ T cells), and elevated VISTA expression, the MOC2 model was selected for further study. Fractionated RT (3Gyx5) was used to mirror clinical HNC protocols.

**Figure 3.**
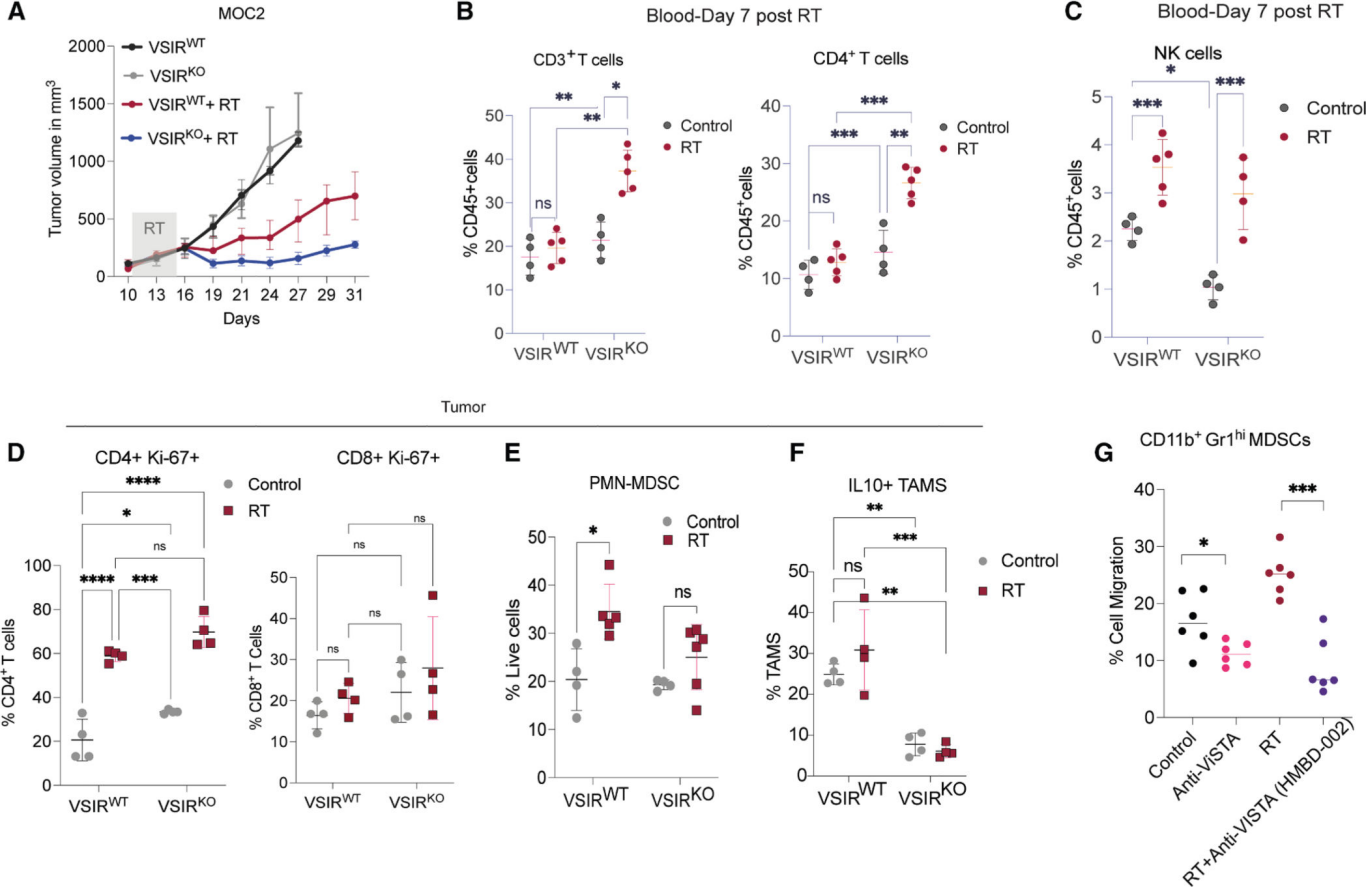
Tumors in *VSIR*^−/−^ mice show enhanced tumor control post-RT treatment (A) Tumor growth curves of MOC2 syngeneic mouse tumors grown in *VSIR*^*WT*^ vs. *VSIR*^*KO*^ hosts with or without 3Gyx5 RT treatment. (B–F) Quantification of early changes (day 7 after start of treatment) in the circulating immune cells in *VSIR*^*WT*^ vs. *VSIR*^*KO*^ mice bearing MOC2 tumors treated with or without RT; *n* = 4–5/group. (B) CD3^+^ and CD4^+^ T cells (percentage of CD45^+^ cells); (C) NK cells (percentage of CD45^+^ cells); (D) proliferating CD4^+^ T cells-Ki-67^+^ (percentage of CD4^+^ T cells) and Ki-67^+^CD8^+^ T cells; (E) PMN-MDSCs (percentage of live cells); (F) IL10^+^ TAMs (percentage of TAM). (G) Splenic CD11b^+^Gr1^hi^ cells were sorted from mice bearing MOC2 tumors treated with different conditions. We seeded 5 × 10^5^ cells in the top chamber of transwell, and the number of MDSCs migrating to the bottom chamber after 4 h was quantified. Two-tailed unpaired t test was used to calculate the *p* values; *p < 0.05; **p < 0.01; ***p < 0.001; ****p < 0.0001. All animal experiments were repeated at least two times, and representative results are shown.

We next investigated the potential of VISTA^+^ myeloid cells in mitigating RT response by examining the changes in MOC2 tumor growth following RT in *VSIR* WT (*VSIR*^*WT*^ *or VISTA*^*+/+*^) and knockout (*VSIR*^*KO*^ *or VISTA*^−/−^*)* mice. MOC2 tumors exhibited consistent growth kinetics regardless of VISTA status in non-irradiated conditions (Figure 3A). However, irradiated tumors in the *VSIR*^*KO*^ mice grew significantly slower than in *VSIR*^*WT*^ mice (Figure 3A), indicating that VISTA mitigates RT efficacy.

One week after initiating RT (see Figure S2B, experimental design), we examined blood samples from the different treatment groups for early systemic immune changes (Figures 3 and S2). In the non-irradiated mice, there was no difference in the baseline CD3^+^ cell count between *VSIR*^*WT*^ and *VSIR*^*KO*^ mice. However, the irradiated *VSIR*^*KO*^ group showed a note-worthy increase (~22%–38%) in total CD3^+^ T cells, primarily due to an increase in CD4^+^ T cells (Figure 3B). While there was a higher level of CD8^+^ T cells in the irradiated *VSIR*^*KO*^ compared to *VSIR*^*WT*^ mice, the difference did not reach statistical significance (Figure S2C). RT increased M-MDSCs in both groups, while PMN-MDSCs remained unchanged (Figures S2D and S2E). These findings suggest dynamic changes in both M-MDSC and T cell populations, with a significant rise in CD4^+^ T cells systemically in *VSIR*^*KO*^ mice following radiation. Natural killer (NK) cell counts, lower basally in *VSIR*^*KO*^, rose similarly post-RT in both groups (Figure 3C).

We next examined the tumors at 2 weeks post-RT. We observed a higher baseline level of proliferating CD4^+^ T cells in tumors growing in *VSIR*^*KO*^ mice, and this effect was further enhanced by RT (Figure 3D). Our results are in line with a recent study, which found that the lack of VISTA on T and myeloid cells in *VSIR*^*KO*^ mice may lead to reduced *cis*- and *trans*-interactions with LRIG1 (the inhibitory receptor of VISTA), resulting in impaired T cell proliferation and survival.^21^

RT-induced PMN-MDSC infiltration was significant in *VSIR*^*WT*^ tumors but blunted in *VSIR*^*KO*^ (Figure 3E), consistent with reports of impaired myeloid cell migration in VISTA-deficient models in response to inflammatory stimuli.^22^ Another observed difference was in levels of interleukin-10-positive (IL-10^+^) TAMs post-RT, which increased in *VSIR*^*WT*^ vs. *VSIR*^*KO*^ mice (Figure 3F). These results suggest that *VSIR* deletion may affect the migration of PMN-MDSC and TAMs, while promoting the recruitment and proliferation of T cells in the TME. To confirm the role of VISTA in MDSC migration, splenic MDSCs from mice bearing MOC2 tumors treated with anti-VISTA, RT, or both were tested in transwell chemotaxis assays. Anti-VISTA blockade alone marginally reduced migration (11% vs. 17% in control), while RT boosted MDSC migration to 25%. Combining RT with anti-VISTA suppressed migration to 9% (Figure 3G), demonstrating the involvement of VISTA in RT-driven MDSC recruitment.

### Single-cell analyses reveal that TAMs and PMN-MDSCs are significantly modulated in response to radiation in *VSIR*^*KO*^ mice

**Figure 4.**
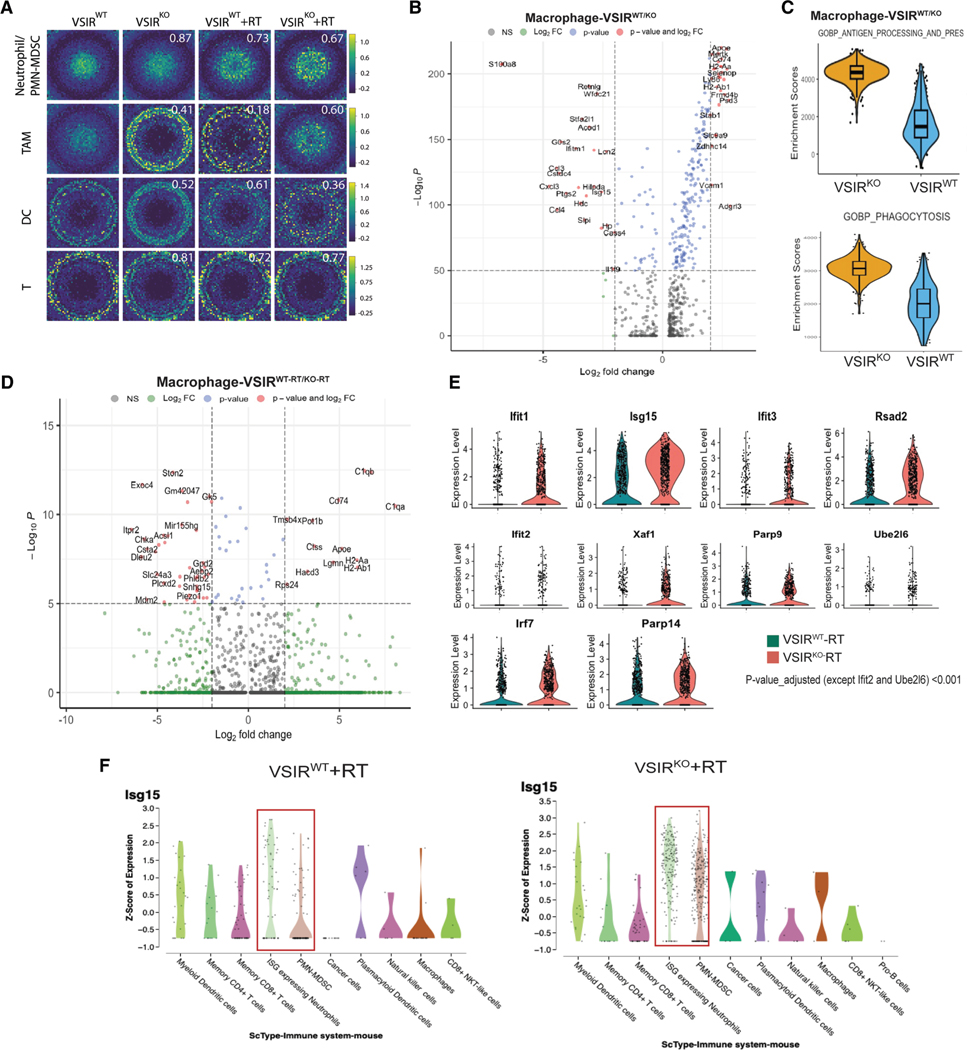
Single-cell RNA sequencing reveals myeloid remodeling response to RT and VISTA (A) Genomap is a computational approach for visualizing scRNA-seq data by transforming the gene count matrix into image representations based on gene-gene interactions, where each pixel corresponds to a specific gene. The figure shows the genomaps of different tumor-infiltrated immune cells (PMN-MDSCs/neutrophils, TAMs, DCs, T cells) from the four groups of tumor-bearing mice (see Figure 3A). Genes with low expression levels are depicted in blue, while those with high expression are shown in yellow. (B) Differentially expressed genes in TAM clusters in the tumors from *VSIR*^*WT*^ or *VISR*^*KO*^ hosts without radiation treatment. (C) Gene enrichment scores showing gene signature scores for antigen presentation (top) and phagocytosis (bottom). (D) Differentially expressed genes in TAM clusters in tumors from the *VSIR*^*WT*^-RT and the *VSIR*^*KO*^-RT groups. (E) Violin plots representing expression levels of various genes in the ISG gene signature obtained from the tumors in the *VSIR*^*WT*^-RT vs. *VSIR*^*KO*^-RT group; *p* adjusted < 0.001. (F) Expression *Z* score of ISG15 across different tumor immune cell types under *VSIR*^*WT*^-RT and the *VSIR*^*KO*^-RT groups analyzed using scRNA-seq data analyzed with Cellenics platform.

To understand how VISTA shapes the immune changes in the irradiated TME, we performed scRNA-seq analysis on MOC2 tumors grown in *VSIR*^*WT*^ and *VSIR*^*KO*^ mice 2 days post-RT dose (3Gyx5) and identified main cell clusters related to the *VSIR*-associated changes with and without RT treatment. Without RT, *VSIR*^*KO*^ tumors showed reduced neutrophils and increased T/NK/dendritic cell (DC) enrichment vs. *VSIR*^*WT*^ (false discovery rate <0.05; Figures S3A and S3B). This aligns with the recent study by Zhang et al.,^23^ showing that VISTA deficiency significantly reduced tumor-associated MDSCs but expanded DCs and enhanced T cell-mediated immunity. To understand the cell types that had the most significant changes under these conditions, we used Genomap, an entropy-based cartography strategy.^24^ Genomap helps visualizing scRNA-seq data by transforming the gene count matrix into image representations based on gene-gene interactions, where each pixel corresponds to a specific gene. Genes with low expression levels are depicted in blue, while those with high expression are shown in yellow. Notably, macrophages exhibited a marked shift in gene expression patterns with *VSIR* deletion, followed by DC. Post-RT, macrophages and the PMN-MDSC/neutrophil clusters showed the largest changes (Figure 4A).^23^

Sub-clustering analyses revealed significant differences in each cell population; however, the incomplete separation between myeloid clusters suggested overlap and highly plastic phenotypes. Macrophage clusters in *VSIR*^KO^ tumors showed significant upregulation of MHC class II molecules (H2Aa, H2Ab1) (Figure 4B) and downregulation of chemokines (CCL3, CXCL3, CCL4), consistent with our impaired monocyte/neutrophil migration data and other reports indicating that VISTA is important to myeloid recruitment (Figure 4B). Notably, one of the most downregulated genes in the tumor in *VSIR*^*KO*^ mice was S100A8, which is an established marker of immunosuppressive macrophages.^25^ Gene set enrichment analyses revealed that both phagocytosis and antigen presentation pathways were significantly enriched in macrophages from the *VSIR*^*KO*^ compared with those from *VSIR*^*WT*^ mice (Figure 4C). Further comparison of the macrophages from *VSIR*^*WT*^ and *VSIR*^*KO*^ tumors post-RT treatment showed three dominant clusters, which we defined as M1-polarized (Adgre1, Nos2, Rsad2, Isg15, Ifit1, Tnf, Ccl6), M2-polarized (Mrc1, Apoe, Lgmn, Il10 Selenop), and C1q macrophages (C1q, C1b, C1c, Apoe, CD74). We observed a significant enrichment of M1-polarized TAM clusters post-RT treatment, with the highest proportion in the *VSIR*^*KO-RT*^. We also saw an increased proportion of C1q-expressing macrophages in the *VSIR*^*KO*^ compared to the *VSIR*^*WT*^ *g*roup (Figures 4C, S3C, and S3D).^26–29^ However, *VSIR*^*KO*^ mice showed reduced complement activation in macrophages post-RT, which may help decrease inflammation-driven tumor progression and treatment resistance. CD74, a survival/proliferation mediator via migration inhibitory factor signaling, was >20-fold lower in *VSIR*^*KO*^, potentially disrupting tumor-promoting pathways.^30,31^

In neutrophils, very few genes showed differential expression, with a cutoff ≥1.5-fold between the WT and the KO groups. Gene set enrichment and pathway analyses in the *VSIR*^*WT-RT/KO-RT*^ revealed enhanced IL-6/STAT3 (signal transducer and activator of transcription 3) and IL-2/STAT5 signaling in *VSIR*^*WT-RT*^ compared to *VSIR*^*KO-RT*^ (Figure S3E). Both pathways can prevent MDSC differentiation and promote proliferation of immature myeloid cells.^32,33^ Zhang et al. recently showed that VISTA deletion impaired STAT3 activation and polyamine biosynthesis, hindering MDSC immunosuppression.^23^ Recent studies highlight that neutrophils with interferon (IFN)-stimulated gene (ISG) signatures are critical for effective immunotherapy.^34,35^ Thus, we evaluated the IFN-15 gene signature in our single-cell analyses. Out of the 15 genes, 5 were excluded from the analyses (3 [Mx1, Gbp1, Apol6] were not represented in the dataset; 2 [Herc5, Ifi6] were not found in mice). Among the 10 IFN genes tested, 8 showed a significant upregulation in *VSIR*^*KO-RT*^ compared to *VSIR*^*WT-RT*^ conditions (Figure 4E), suggesting that neutrophils in the *VSIR*^*KO-RT*^, compared to those in the *VSIR*^*WT-RT*^ mice, have higher IFN gene expression indicative of a therapy-responsive population. ISG15 expression was highest in PMN-MDSCs and ISG-induced neutrophils and significantly elevated in *VSIR*^*KO-RT*^ versus *VSIR*^*WT-RT*^ (Figure 4F). Although we saw some enrichment of IFN-responsive signature within the macrophage subsets, it was not significant. Overall, our data indicate that VISTA deletion combined with RT repolarizes myeloid cells by activating type I IFN responses, enhancing antigen presentation and phagocytosis.

### VISTA blockade improves RT responses in HNC and other models

**Figure 5.**
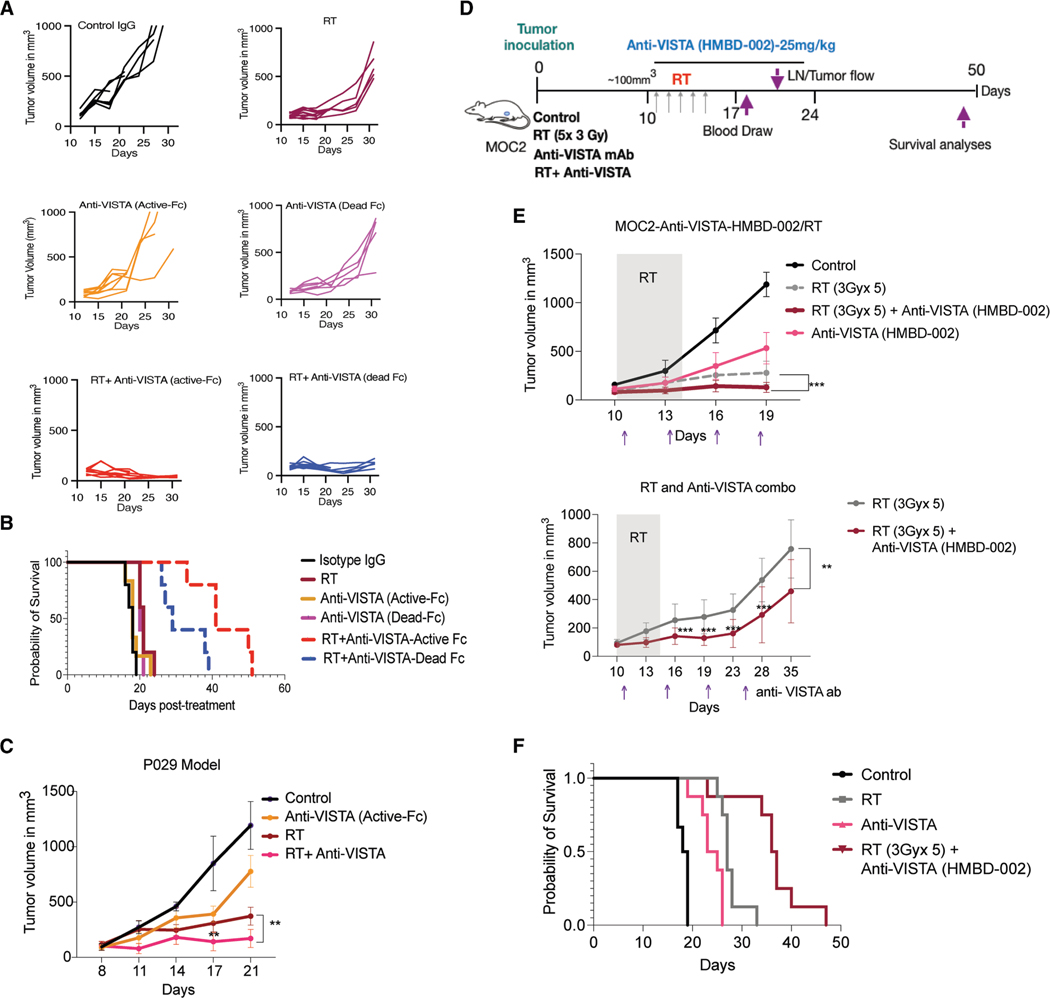
VISTA blockade significantly enhances RT response in the head and neck syngeneic mouse tumor model (A) Individual tumor growth curve of MOC2 tumors treated with control IgG, RT alone, anti-VISTA antibody alone (either with an active Fc or with a dead Fc [LALA/PG mutations in the mIgG2a isotype]), or RT with anti-VISTA antibody. (B) Kaplan-Meier survival curve of MOC2 tumor-bearing mice with different treatment regimens (*n* = 5–6). Death was determined as the time mice needed to be euthanized due to very large or ulcerated tumors. (C) Tumor growth curves showing the response of P029 tumors in C57/BL6 mice treated with either RT alone (3Gyx5), anti-VISTA antibody (SG7-active Fc) alone, or RT + anti-VISTA antibody (SG7-active Fc) (*n* = 5 mice/group). (D) Treatment schema for combination therapy of RT and HMBD-002 anti-VISTA antibody. For the combination treatment, the tumors were irradiated with 3Gyx5 fractions once they reached 100 mm^3^. Isotype or anti-VISTA (HMBD) antibodies were injected concurrently every 4 days, starting on the first day of RT treatment, for a total of six doses. (E) (Top) Average tumor volume over time for the different treatment groups in (D). (Bottom) Growth curves of the RT and RT + anti-VISTA groups from day 10 through day 35. The other two groups, control and anti-VISTA alone, had to be sacrificed before days 20 and 30, respectively, due to very large or ulcerated tumors. (F) Kaplan-Meier survival curve of MOC2 tumor-bearing mice in different treatment groups (*n* = 5–6). Death was determined as the time mice needed to be euthanized due to very large or ulcerated tumors. Tumor volumes were assessed using two-way ANOVA, while mouse survival rates were examined with the Kaplan-Meier method and compared through log rank tests. *p < 0.05; **p < 0.01; ***p < 0.001; ****p < 0.0001.

We next determined whether the antibody-mediated blockade of VISTA would lead to enhanced tumor control with RT. We used a conventionally fractionated RT (3Gyx5) in combination with an anti-VISTA (SG7) containing an ‘‘active Fc’’ domain that is competent to engage Fcγ receptor (FcγR)-mediated effector functions (mIgG2a isotype) or one with a ‘‘dead Fc’’ domain (LALA/PG mutation) used to prevent depletion of healthy VISTA-expressing cells and allow the parsing of antibody-mediated VISTA blockade.^14^ While both SG7 antibodies by themselves had a negligible effect on tumor growth inhibition (Figure 5A), the combined treatment of RT and anti-VISTA significantly inhibited tumor growth with both molecules (active or dead Fc) (Figure 5A). The combined treatment also led to significantly longer overall mouse survival, with a median survival of 55 days for the anti-VISTA (active Fc)/RT combination, 39 days for the anti-VISTA (dead Fc)/RT combination, and 24 days for RT alone (Figure 5B). These data suggest that the active Fc component prolonged tumor control duration when combined with RT. Similar results were observed in the P029 HNC model, where RT and anti-VISTA (active Fc) treatment showed significant tumor growth inhibition compared to the individual treatment groups (Figure 5C). However, previous trials showed that a VISTA antibody with an Fc-dependent activity of the IgG1 isotype, despite its anticancer activity, triggered substantial cytokine release, leading to neurotoxicity at subtherapeutic doses (NCT04475523). Recently, another VISTA-targeting antibody that is being evaluated in early-phase clinical trials has been shown to block VISTA and induce an antitumor response without requiring Fc-dependent IgG1 isotype activity^36^ (Hummingbird Bioscience, HMBD-002). Since this is a clinical-grade antibody that would allow rapid translation to the clinics with RT, we tested HMBD-002 with RT in our preclinical models. We started the RT treatment post-randomization of mice at ∼100 mm^3^ and dosed either isotype IgG4a control or HMDB002 biweekly at 25 mg/kg for 6 doses (Figure 5D). Median survival reached 35.5 days (combination) vs. 27 days (RT alone) and 24 days (HMBD-002 alone) (Figures 5E and 5F). HMBD-002 monotherapy showed transient 40%–50% growth inhibition, but tumors rebounded post-treatment, while RT combination sustained delay.

We also tested the combination of RT + HMDB002 in two other tumor models (B16BL6 melanoma and 4T1 breast cancer). An ablative RT dose (12Gyx2) was used for these experiments (Figure S5A). RT + HMDB002 led to the longest tumor growth delay for both models, although the difference from RT alone was small, likely due to the larger dose of RT used here (Figures S5B and S5C). These data confirmed that irrespective of the antibody types, VISTA blockade could potentiate RT responses in tumors independent of the antibody-dependent cellular cytotoxicity-mediated effects.

### Combination of VISTA blockade with radiation induces a robust systemic antitumor immune response

**Figure 6.**
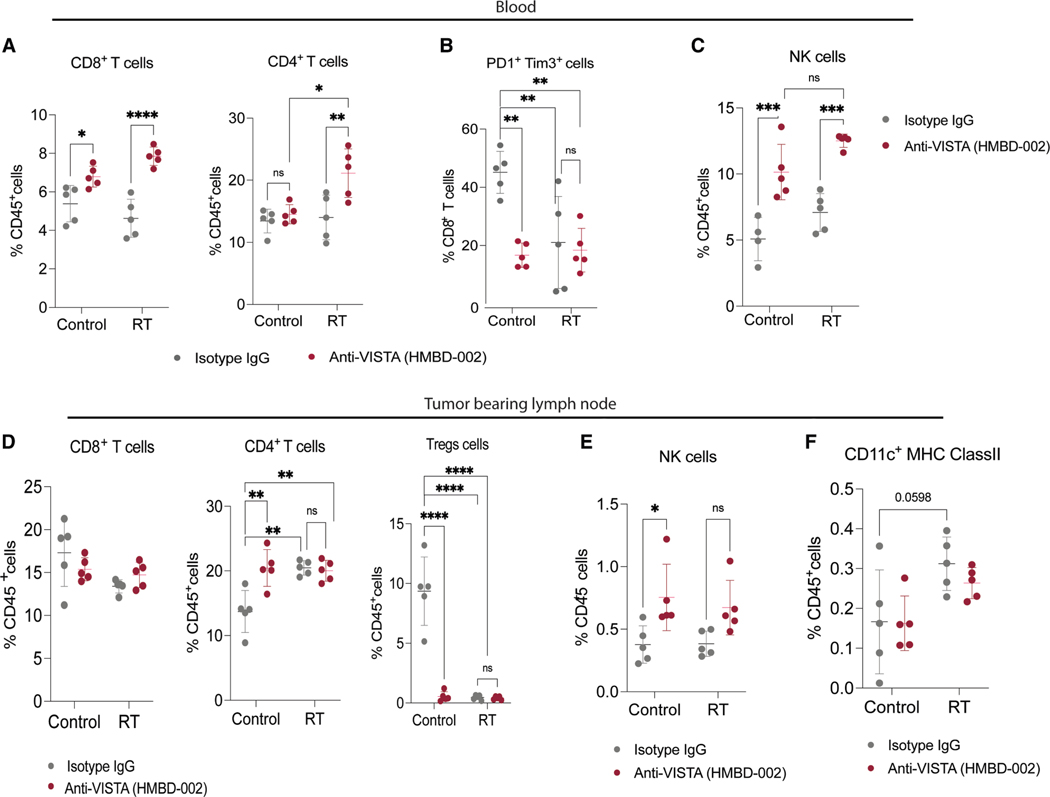
Combination of VISTA blockade with radiation induces robust systemic antitumor immune response (A–C) Level of different immune cell types in the blood of MOC2 tumor-bearing mice at 1 week from the start of the indicated treatments as determined by flow cytometry (*n* = 5): (A) T cells (CD8^+^ and CD4^+^ T cells) as a percentage of circulating CD45^+^ cells; (B) PD1^+^Tim3^+^CD8^+^ T cells as a percentage of circulating CD8^+^ T cells; (C) NK cells as a percentage of circulating CD45^+^ cells. (D and E) Level of different immune cell types in the tumor-draining lymph nodes of tumor-bearing mice at 2 weeks from the start of the indicated treatment: (D) CD8^+^ T cells, CD4^+^ T cells, and Treg cells (CD4^+^FoxP3^+^) as a percentage of CD45^+^ cells; (E) NK cells as a percentage of CD45^+^ cells. (F) Conventional dendritic cells (CD11c^+^MHC class II^+^) as a percentage of CD45^+^ cells. *p < 0.05; **p < 0.01; ***p < 0.001; ****p < 0.0001.

To elucidate the mechanism that inhibits tumor growth in the combined treatment, we conducted an early assessment of systemic changes. On day 7, following RT initiation and anti-VISTA treatment (Figure 5D), we observed an increase in CD8^+^ T cells in mice treated with anti-VISTA monotherapy (Figure 6A). Conversely, the RT group showed no alteration in CD4^+^ and CD8^+^ T cell numbers at this time point (Figure 6A). In contrast, the combination group exhibited a significant increase in both cell types compared to the monotherapy groups and vehicle controls (Figure 6A). We also explored PD-1 and Tim-3 expression on CD8^+^ T cells as exhaustion markers. Results revealed that nearly 40% of CD8^+^ T cells in the control group expressed PD1 and Tim-3, which was reduced to half with anti-VISTA treatment alone (Figure 6B). RT alone led to a moderate reduction in this population, while the combination group showed a 50% decrease in exhausted CD8^+^ T cells, suggesting that VISTA inhibition predominantly drove this response in MOC2 tumors.

We also examined NK cell responses under these conditions. Anti-VISTA treatment increased the percentage of NK cells in the blood (from 5% in the control to 10%; Figure 6C), whereas RT did not significantly alter this number. In contrast, the combination treatment showed an increase of ∼13% (Figure 6C).

We next evaluated the treatment changes in the tumor-draining lymph nodes, which are crucial for initiating and regulating antitumor immunity (refer to the schema in Figure 5D). Although no difference in CD8^+^ T cells was observed, there was a significant but similar increase in CD4^+^ T helper cells with anti-VISTA treatment alone, RT alone, or combined treatment (Figure 6D). The most pronounced difference was seen in CD4^+^FoxP3^+^ Tregs, which were reduced to near-undetectable levels with either monotherapy or combination therapy (Figure 6D). This aligns with prior studies highlighting the role of VISTA in Treg differentiation and function.^37^ We also noted moderately enhanced NK cell numbers, primarily with anti-VISTA treatment (Figure 6E). Given the critical role of DCs in priming and regulating T cell function in lymphoid tissues, we assessed whether the treatments affected DC numbers in the lymph nodes. An increasing trend was observed in the RT-treated mice, which was maintained in the combination group (Figure 6F). Overall, our findings indicate that the combination treatment of anti-VISTA and RT induces immune changes in the blood and draining lymph nodes that can potentiate antitumor immunity.

### The combination of VISTA blockade with radiation enhances T cell function and antitumor immunity through myeloid repolarization

**Figure 7.**
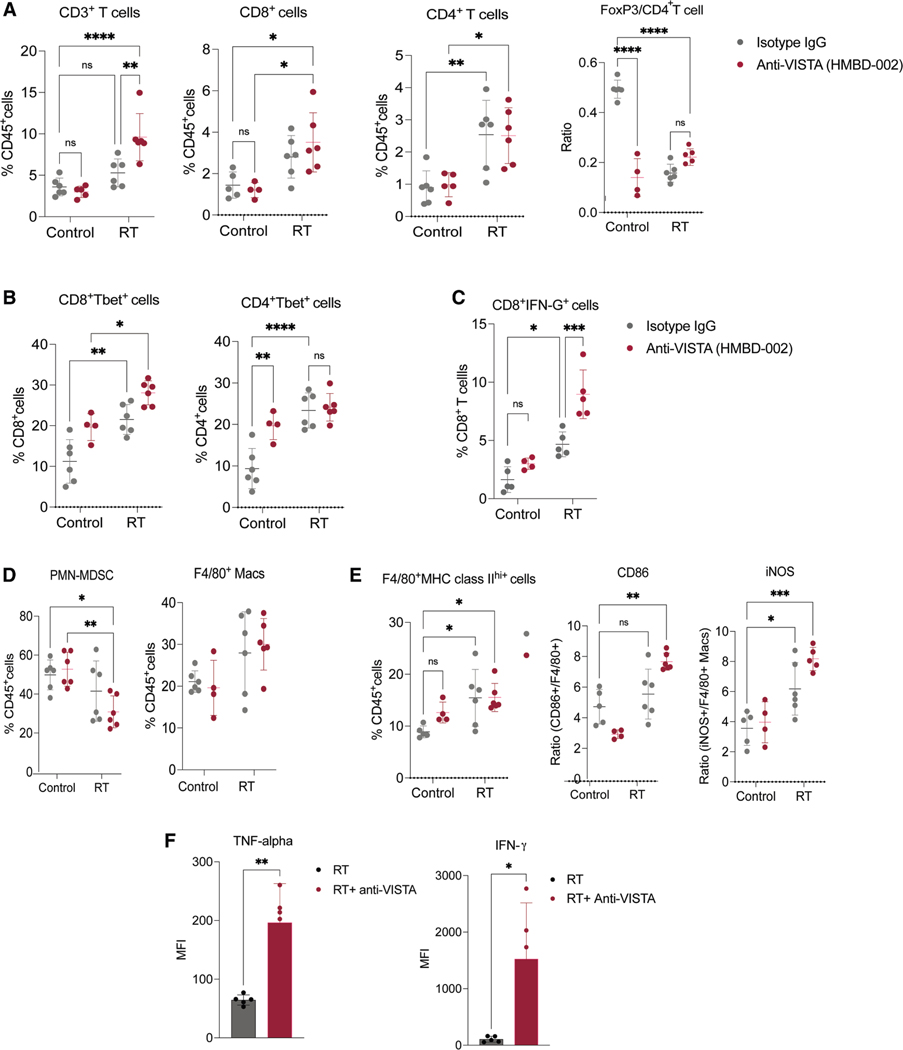
Combination of VISTA blockade with radiation enhances T cell function and antigen presentation in the tumor (A) Level of different T cell types (CD3^+^, CD8^+^, CD4^+^ T cells as a percentage of CD45^+^ cells and the ratio of FoxP3^+^ Treg/CD4 T cells) in the tumor at 2 weeks from the start of the indicated treatment (*n* = 4–6). (B) Percentage of T-bet^+^ cells among CD8^+^ and CD4^+^ T cells in the tumors at 2 weeks from the start of the indicated treatment (*n* = 4–6). (C) Percentage of IFN-γ^+^ cells among CD8^+^ T cells in the tumors at 2 weeks from the start of the indicated treatment (*n* = 4–6). (D) Level of PMN-MDSCs and F4/80^+^ macrophages as a percentage of CD45^+^ cells in the tumor at 2 weeks from the start of the indicated treatment (*n* = 4–6). (E) Quantitation of MHC class II and other macrophage maturation markers (CD86 and iNOS) on F4/80^+^ macrophages in the tumors at 2 weeks from the start of the indicated treatment (*n* = 4–6). (F) Luminex data showing levels of tumor necrosis factor α and IFN-γ secreted cytokines in the plasma of mice (*n* = 5/group) bearing MOC2 tumors treated with RT (3Gyx5) or RT+ anti-VISTA antibody at 2 weeks from start of treatments. *p < 0.05; **p < 0.01; ***p < 0.001; ****p < 0.0001.

We next assessed the immune microenvironment in the tumor 2 weeks after treatment initiation to better understand the effect of each therapy. In the anti-VISTA + RT arm, we observed the highest increase in the total CD3^+^ T cells within the tumor compared to individual treatments and untreated controls. This increase encompassed both CD4^+^ and CD8^+^ T cell subsets (Figures 7A and S4). Despite no obvious change in the total CD4^+^ T cells with anti-VISTA or RT treatment alone, the FoxP3/CD4^+^ T cell ratio significantly decreased in the group treated with anti-VISTA alone, suggesting an impact on Treg differentiation or recruitment within the tumor. We saw a similar trend in the RT-treated group, which was also maintained in the combination arm (Figure 7A).

Examining T-bet, a crucial regulator of T helper 1 (Th1) cell generation, effector differentiation, and function in tumor-infiltrating lymphocytes, we noted a significant increase in Tbet^+^ cells in CD8^+^ and CD4^+^ T cells post-treatment. This increase was most pronounced in the combination group, with CD8^+^Tbet^+^ cells rising to 28% compared to 11% in the control group, and CD4^+^Tbet^+^ cells rising to ~22% from 10% (Figure 7B). Likewise, the proportion of CD8^+^IFN-γ^+^ cells increased approximately 4-fold with the combined treatment compared to untreated controls (Figure 7C for MOC2 tumor, Figure S5D for 4T1 tumors), indicating a strong Th1 differentiation phenotype with anti-VISTA and RT.

Since VISTA is highly expressed in myeloid cells and our data reveal a strong link between VISTA and myeloid lineage, we investigated the changes in different myeloid cell subsets with treatment. Although anti-VISTA alone did not result in a change in the PMN-MDSC level, RT did reduce the number of these cells in the tumor, and the combined treatment led to the largest reduction (Figure 7D). In the 4T1 tumors, RT alone led to an increase in M-MDSC, whereas the addition of anti-VISTA led to a reduction of these cells to baseline (Figure S5D). Similarly, while anti-VISTA treatment alone did not affect F4/80^+^ macrophage numbers, RT and RT + anti-VISTA led to an increase in F4/80^+^ TAMs (Figure 7D). Further analysis of macrophage markers, including the expression of MHC class II CD86 and inducible nitric oxide synthase (iNOS), revealed that RT alone enhanced M1 marker expression, suggesting a shift in macrophage polarization (Figure 7E). This M1 polarization was even more pronounced in the combination group, with nearly 3-fold induction compared to untreated controls. Luminex analyses of plasma samples from mice treated with RT alone or RT + anti-VISTA confirmed a significant increase in tumor necrosis factor α and IFN-γ levels in the combined treatment group compared to RT alone (Figure 7F). Overall, these findings indicate that the combination of RT and anti-VISTA results in a broad immune activation, particularly changes in effector T cells and macrophage polarization, triggering a robust antitumor immune response within the tumor.

## DISCUSSION

Our study provides insights into the intricate interactions between VISTA expression on myeloid lineage cells and their impact on the TME, particularly in response to RT. Through a combination of *in vivo* mouse models and analysis of patient samples, the study highlights the significance of targeting VISTA-mediated immunosuppression to enhance the therapeutic efficacy of RT.

Our data validate recent studies^19,38–40^ that reported an increase in the presence of immunosuppressing cells like TAMs and MDSCs in the circulation and TME with fractionated RT. We showed that RT increased VISTA expression in MDSCs, particularly PMN-MDSCs, leading to a higher number of these cells in the TME and systemically in mouse tumor models and patients with HNC. Moreover, our study demonstrates that either genetic deletion of VISTA in the host or its blockade with different antibodies leads to enhanced HNC control by RT and prolonged survival in tumor-bearing mice, suggesting that targeting VISTA could overcome RT resistance mediated by myeloid cell immunosuppression. This is consistent with previously published data in mice bearing Lewis lung carcinoma, B16F10 melanoma, or MC38 colon cancer treated with single-fraction RT,^23,41^ as well as in a preclinical breast cancer model.^42^

Here, we explored further mechanistic insights into how VISTA deletion or blockade enhances RT by evaluating the tumor, nodal, and systemic responses to the treatment. We found that combined VISTA blockade with RT not only affects the TME by repolarizing myeloid cells and enhancing effector T cell number and function but it also induces a robust systemic antitumor immune response in the blood and lymph nodes characterized by more circulating T cells, less exhausted T cells, more NK cells, fewer Treg cells, and more DCs in the draining lymph nodes. This is consistent with previously published data showing that the combination of RT and VISTA blockade resulted in reduced accumulation of both peripheral and tumor-infiltration tumor-associated neutrophils, M-MDSC with concomitant expansion of activated CD8^+^ T cells.^23^

Our scRNA-seq study from the tumor provides further mechanistic insights into the underlying response to RT and VISTA deletion within the TME. We note significant alterations in the gene expression profile of macrophages and PMN-MDSCs, specifically in immune-activating signatures and chemokine expression. Recent literature suggests that the complement pathway is activated post-RT and that it is associated with poor prognosis.^28,29^ The radiotherapeutic efficacy was significantly enhanced in mice cotreated with tumor-targeted complement inhibition; this effect was associated with early increases in the number of apoptotic cells and increased inflammation.^28^ Our results showed that the combination treatment led to a significant downregulation of C1q macrophages, which are associated with immunosuppressed TME, characterized by high expression of immune checkpoints.

PMN-MDSCs and monocytic MDSCs are known to be strongly pro-tumorigenic and immunosuppressive.^43^ However, some studies suggest that certain myeloid cell subsets are associated with antitumor activity.^44^ Therefore, myeloid cells with similar surface molecules can exhibit different func-tions.^34,45^ Through our single-cell data, we observe that the neutrophil lineage shows significant plasticity with different gene expression profiles, especially post-RT treatment in the *VSIR*^*WT/KO*^ groups. We see enhanced ISG signatures in neutrophils from the TME of *VSIR*^*KO*^ compared to *VSIR*^*WT*^ mice post-RT treatment. This is congruent with recent reports suggesting that IFN-stimulated neutrophils positively predict ICI outcomes and are functionally involved in the generation of response.^35^ Thus, our data suggest that VISTA blockade with RT promotes a shift in myeloid cell polarization toward an antitumor phenotype, characterized by enhanced antigen presentation and type I IFN responses.

A unique aspect of our study involves testing observed effects using different anti-VISTA antibodies: a pair of antibodies (SG7) with the same VISTA binding epitope but with either an active Fc or a silent/dead Fc, or HMBD-002,^36^ which has subnanomolar affinity to mouse VISTA and contains an IgG4 Fc domain that does not mediate Fc-dependent activity. There are some additional differences to note about the antibodies used in this study: SG7 and HMBD-002 bind to distinct epitopes on VISTA^12^ and thus may block different subsets of VISTA ligands. While all three antibodies enhanced the anti-tumor activity of RT, a comparison of the matched SG7 antibodies suggests that an active Fc that engages FcγR may be more efficacious compared to an antibody lacking Fc activity. However, since Fc activity has been linked to some clinical toxicity noted above, a VISTA antibody that optimizes tumor activity while mitigating toxicity is critical for the clinical success of targeting this pathway. Toward this goal, in addition to HMBD-002, other anti-VISTA antibody programs, including KVA12123 (Kineta) containing an engineered IgG1 with a YTE mutation for longer half-life and potentially reduced FcγR activity,^36,46^ have not shown evidence of cytokine release syndrome or dose-limiting toxicity in humans.^46,47^ Given its broad expression on immune cells, other approaches to VISTA blockade have focused on conditionally active pH selective targeting of anti-VISTA antibodies to the acidic TME, including Sensei SNS-101^12^ (Sensei BioTherapeutics) and BMS767^8^; SNS-101 has been shown to be well tolerated in humans, with no dose-limiting toxicities observed.

The strengths of the study include its in-depth assessment of the mechanism involved in the synergism between VISTA blockade and RT and the use of different antibodies targeting this pathway. A major weakness of our study is that while we have characterized the different immunomodulatory myeloid subtypes after RT, we have not fully explored the functional consequences of these changes and cannot conclusively attribute the observed therapeutic effects to a specific cell type without targeted depletion studies. This limitation leaves room for further investigation into how the observed altered gene expression profiles in myeloid subsets would translate to changes in their immunosuppressive capabilities. While we included patients with HPV^+^ and HPV^−^ tumors in this study, our preclinical models primarily focus on HPV^−^ tumors. Hence, we cannot determine the relationship between VISTA expression and HPV status. Our limited focus on HPV^−^ HNC models was primarily due to the enrichment of immunosuppressive myeloid cells (TAMs and MDSCs) in their TME as well as their poor outcomes when treated with RT. Our findings suggest that targeting myeloid checkpoints offers a promising approach to enhance the efficacy of RT in myeloid-rich tumors. These results lay the ground-work for future investigation of this strategy across a broader spectrum of cancer types.

In summary, our study sheds light on the complex interplay between VISTA expression on myeloid cells and the antitumor immune response to RT. Targeting VISTA-mediated immunosuppression emerges as a promising strategy to enhance the efficacy of RT and overcome treatment resistance in patients with HNC. Future clinical studies are warranted to validate these findings and explore the translational potential of combining VISTA blockade with RT in the management of HNC as well as other solid tumors.

### Limitations of the study

While the study employs multiple preclinical models, their use in a subcutaneous setting limits physiological relevance, as they may not fully recapitulate the immune and TME heterogeneity seen in human tumors. The orthotopic HNC buccal models present significant challenges for radiation combination experiments due to functional (compromised feeding behavior leading to reduced observation windows for radiation responses) and methodological (focused radiation delivery and imaging requirements) constraints. The second limitation in our study is that while we have characterized the different immunomodulatory myeloid subtypes after RT, the absence of targeted myeloid cell depletion experiments prevents definitive attribution of therapeutic effects to specific cell populations.

## RESOURCE AVAILABILITY

### Lead contact

Request for further information, resources, and reagents should be directed to and will be fulfilled by the lead contact, Quynh Thu Le (qle@stanford.edu).

### Materials availability

This study did not generate new unique reagents.

### Data and code availability

- Data: raw and processed scRNA-seq data have been deposited in the NCBI Gene Expression Omnibus at GEO: GSE295914 and are publicly available. The accession number is also listed in the key resources table.
- Code: all custom scripts and analysis pipelines used in this study have been deposited in Zenodo and are publicly available at: https://doi.org/10.5281/zenodo.15192114. The link is also listed in the key resources table.
- Other: any additional information required to reanalyze the data reported in this paper is available from the lead contact upon request.

## STAR★METHODS

### EXPERIMENTAL MODEL AND STUDY PARTICIPANT DETAILS

#### Animal experiments

For *in vivo* studies, P029 cell line (provided by XJ Wang at the University of Colorado) was cultured in DMEM-F12 medium supplemented with 10% fetal bovine serum (FBS) and 1% antibiotics. MOC2 cells were cultured in a 1:2 mix of DMEM-F12/IMDM with 5% fetal calf serum (FCS) and growth factors, as previously described.^48^ B16L6 cells were purchased from Acceligen (#ABC-TC0060) and 4T1 cells from ATCC (#CRL-2539) and grown according to the manufacturers’ recommendations. All cell lines have been authenticated by STR fingerprinting and were confirmed to be free of mycoplasma using the Mycoalert Mycoplasma Detection Kit (Lonza, L-T07–318) before experimental use.

#### *In vivo* experiments

All mice were handled and euthanized consistent with the ethics guidelines and conditions set and overseen compliance with the Administrative Panel on Laboratory Animal Care (APLAC) guidelines and approved by the IACUC of Stanford University, under APLAC protocol #15106 (Research Compliance Office, Palo Alto, California, USA). Seven- to nine-week-old C57BL/6 and Balb/c mice were purchased from Charles River Laboratories. To generate subcutaneous tumors, 2.5×10^5^ (MOC2, B16L6, 4T1) cells or 5×10^5^ P029 cells were injected into the right flank region of the mice. Tumor growth was measured every 3 days using a vernier caliper until euthanasia. Mice were randomized into groups, with treatment beginning when tumor volume was ∼100–150 mm^3^. Mice were euthanized, and tumors were removed along with the spleen, draining LNs, and lungs. Tissues used for immunohistochemical analysis were fixed with 10% neutral buffered formalin (NBF) overnight and then stored in 70% ethanol until paraffin fixation. Tissues for flow cytometric analysis were collected in RPMI media and processed immediately. LNs and lungs were analyzed for metastases using H&E staining.

VISTA whole body knock-out mice (*VSIR*^−/−^ mice) embryos generated on a C57BL/6N background were obtained from Mutant Mouse Regional Resource Centers (www.mmrrc.org; stock no. 031655-UCD; B6;129S5-Vsirtm1Lex/Mmucd) as cryopreserved embryos, which were rederived at Stanford Animal Facility. Upon rederivation, we crossed the original *VSIR*−/− mice with C57BL/6N mice for one generation and inter-crossed heterozygous off-springs to obtain *VSIR KO* and WT littermates. All experiments used sex and age-matched mutant mice and WT littermates. The mice used in the experiments were verified for genotypes further by PCR genotyping.

#### Treatment regimens

Both the SG7 and dead-Fc anti-VISTA antibodies, developed by the Cochran lab, were administered at 20 mg/kg twice weekly, starting with the first dose of radiation therapy (3 Gy×5 for MOC2 and P029 tumors, 12 Gy×2 for B16L6 and 4T1) for a total of six doses. Anti-VISTA (HMBD-002) antibodies were provided by Hummingbird Biosciences Inc, Singapore. These antibodies, or their isotype controls, were administered biweekly via intraperitoneal (IP) injection at 25mg/kg, starting concurrently with the first dose of RT, for 6 doses for MOC2 and P029 tumors. For B16L6 and 4T1 tumors, an Anti-VISTA (HMBD-002) antibody was administered IP at 25 mg/kg biweekly starting 1 day after RT completion, going forward.

#### Human samples

Samples of peripheral blood and tumor tissues were collected from patients at Stanford Hospital, Stanford, CA. The study was approved by the Stanford Institutional Review Board (IRB protocol 10564 for the blood collection and IRB protocol 55606 for the tissue collection). All patients signed approved consent forms for the study. The patient demographics are provided in Tables S1 and S2.

0.5–1 mL of peripheral blood collected by venipuncture into EDTA-coated Vacutainer™ tubes was used for flow analyses. The whole blood sample was lysed with ACK RBC lysis buffer (incubating for 5–7 minutes) followed by 2x washes with HBSS and then stained with cell surface marker antibodies. All processing was done within 2–4 hours of collection. Human tumors were first digested with human tumor dissociation kit (Miltenyi) and then ACK lysis buffer was used to lyse RBCs, followed by filtering through 70 μm filters to get a single cell suspension.

### METHOD DETAILS

#### Tissue processing and flow cytometry

##### MOC2 and P029 tumor models

Blood samples (50–100 μl) were collected via retro-orbital bleeding using EDTA tubes at various time points. After treatment with ammonium chloride potassium (ACK) buffer to eliminate red blood cells, the samples were stained with an antibody cocktail.

Lymph nodes were crushed on a 70-μm strainer using a syringe plunger into fluorescence-activated cell sorting (FACS) buffer (1× phosphate-buffered saline and 0.5% BSA + 5 mM EDTA). Tumors were minced into 1mm chunks and treated with a tumor dissociation cocktail from Miltenyi. Cells were pelleted (1300 rpm, 5 min, 4°C), lysed with ACK buffer (Gibco; #A1049201), washed twice, and resuspended in FACS buffer. For flow cytometry, 2×10^6^ cells were stained using Zombie NIR Fixable Viability Kit (BioLegend, #423106) for 10 minutes at 4°C, and Fc receptors were blocked with TruStain FcX (anti-mouse CD16/32) antibody (Biolegend, #101320) for 10 minutes at 4°C. After which, cells were stained with fluorophore-conjugated antibodies for lineage and activation markers of immune populations for 30 minutes at 4°C in the dark. Intracellular staining (IC) was performed after surface staining using FIX & PERM Cell Permeabilization Kit (ThermoFisher, #GAS004) per the manufacturer’s protocol. Cell surface staining was performed using fluorophore-conjugated anti-mouse CD45.2 (30-F11, Cat:103147), TCR-beta (H57–597), CD4 (GK1.5, #100411), CD8 (53–6.7, #100712), NK1.1 (PK136), CD11b (M1/70, #101206), CD11c (N418, #117333), MHCII (M5/114.15.2), Ly6G (1A8, #127607), Ly6C (HK1.4, #128041), F4/80 (BM8, #123131) from Biolegend. For intracellular staining, cells were fixed using the Fixation-Permeabilization kit protocol (#88–8824-00, eBioscience) followed by intracellular staining for CD206 (MMR, #141720, Biolegend), Arginase-1 (#17–3697-80; Invitrogen), IFN-Gamma (#505808, Biolegend), and IL-10 (#505028, Biolegend). No *ex vivo* stimulation was performed for detection of the cytokines. We used the following gating strategy to identify different MDSC subsets: G-MDSC (CD11b^+^Ly6G^+^Ly6C^−^), M-MDSC (CD11b^+^Ly6C^+^Ly6G^−^), CD4^+^ T cells (CD45^+^TCR-beta^+^CD4^+^), Tregs (CD45^+^TCR^−^beta^+^CD4^+^FOXP3^+^), CD8^+^ T cells (CD45^+^TCR-beta^+^CD8^+^), NK cells (CD45^+^TCR-beta^−^NK1.1^+^), cDCs (CD11b^+^Ly6G^−^CD11c^+^MHC-class II^+^) and total macrophages (CD11b^+^Ly6G^−^F4/80^+^). Flow cytometry data were acquired using a BD LSR II flow cytometer and analyzed using FlowJo software (Tree Star).

##### B16L6 and 4T1 tumor models

Following harvest, single-cell suspensions were generated by digesting finely cut tumors in RPMI-1640 (ThermoFisher, #11875093) containing 0.1 mg/ml of DNase I (Sigma, #11284932001) and 1 mg/ml Collagenase (Sigma, #1108866001) for 30–45 minutes at 37°C and 100 rpm. The cell suspension was then strained using 40 μm cell strainers (Corning, #352340), and flow through containing cells was subjected to RBC lysis using Red Blood Cell Lysis Solution (Miltenyi, #130–094-183) as per manufacturer’s protocol. Cells were harvested, washed, and counted using a hemocytometer.

For flow cytometry, 3×10^6^ cells were stained using Zombie NIR Fixable Viability Kit (BioLegend, #423106) for 15 minutes at 4°C, and Fc receptors were blocked with TruStain FcX (anti-mouse CD16/32) antibody (Biolegend, #101320) for 10 minutes at 4°C. Cells were then stained with fluorophore-conjugated antibodies for lineage and activation markers of immune populations for 30 minutes at 4°C in the dark. IC staining was performed after surface staining using FIX & PERM Cell Permeabilization Kit (ThermoFisher, #GAS004) as per the manufacturer’s protocol. Cells were then washed and resuspended in 200 μL of FACS buffer (PBS + 0.5% BSA + 2 mM EDTA) for flow cytometric data acquisition on Cytek^®^ Northern Lights 3000 System. Raw data was analyzed using FlowJo 10.8.1 analysis software (Tree Star, San Carlos, CA). Fluorescence minus one (FMO) controls were used for gating.

Cells were incubated with Human FcR Blocking Reagent (MACS™; Miltenyi Biotec) prior to the addition of antibodies directed against cell surface antigens with pre-determined concentrations of antibodies for 20 min at 4°C and were washed twice with FACS buffer. We used the following fluorophores to identify different cell populations: MDSC, CD14 (# 555399, clone M5E2), CD15 (#562371, clone W6D3), CD33 (#340474, clone P67.6), HLA-DR (#339194, clone L243), CD11b (#557743, clone ICRF44), and LOX-1 (#358610, clone 15C4), all from BD Biosciences except Lox-1, which was from BioLegend. For intracellular staining, cells were fixed and permeabilized using fixation/permeabilization (BD Biosciences) solution, stained for 30 min with Arg-1 (R16–715, E-Biosciences), and then resuspended with FACS buffer. We used the following gating strategy for MDSC subsets in PBMC: G-MDSC (CD15^+^CD11b^+^Lox-1^+^), M-MDSC (CD14^+^CD11b^+^HLADR^−^).

#### Single-cell RNA sequencing

Sequencing results were mapped to the mm10–2020-A mouse reference genome using 10X Genomics Cell Ranger 7.0.1.^49^ Analysis was done in R with Seurat 4.1.3.^50^ Gene expression was Log Normalized, and then cells were pooled from duplicate samples amongst like conditions, including *VSIR*^*WT*^ (n = 2298, n = 2097), *VSIR*^*KO*^ (n = 2119, n = 2443), *VSIR*^*WT*^ + RT (n = 382, n = 804), and *VSIR*^*KO*^ + RT (n = 679, n = 503). The top 2000 highly variable features were identified and then gene expression was scaled. Principal component analysis was performed utilizing the highly variable features and then cells were clustered and represented with Uniform Manifold Approximation and Projection (UMAP) plots.

Genomap analyses was run as described in Islam et al., 2023.^24^ Each pixel in the Genomap corresponds to a single gene. The relative positions of these genes on the map reflect their level of interaction, with genes exhibiting stronger interactions placed closer to the center and those with weaker interactions positioned toward the periphery.

For analysis of cell proportions, the *VSIR*^*WT*^ and *VSIR*^*KO*^ and *VSIR*^*WT*^ + RT and *VSIR*^*KO*^ + RT datasets, respectively, were integrated with Harmony.^51^ Gene ontology analysis was performed using the Single Cell Pathway Analysis (SCPA) package and the Hallmark pathways for Mus musculus obtained from the Molecular Signatures Database.^52^ The easy single-cell analysis platform for the enrichment (escape) package was used to perform gene set enrichment analysis (GSEA) for specific cellular processes.^53^ The Enhanced Volcano and dittoSeq packages were used for the visualization of data. The scPropotionTest package was utilized to calculate proportions of cell types in various conditions.^54^

#### *In vitro* chemotaxis assays

For the chemokine-induced migration assay, we utilized splenic myeloid-derived suppressor cells (MDSCs) isolated from mice bearing MOC2 tumors. Single-cell suspensions were prepared by mashing the spleens at D14 post-tumor inoculation through a 70 μm cell strainer, followed by red blood cell lysis using ACK buffer. MDSCs were isolated using a commercial MDSC isolation kit (Miltenyi Biotec) according to the manufacturer’s instructions. The migration assay used 24-well Transwell plates with 3 μm pore size inserts (Corning). The lower chambers were filled with 700 μL of 0.5% FBS containing RPMI-1640 medium containing CCL3 (50 ng/mL). Isolated MDSCs were resuspended in serum-free RPMI-1640 at a concentration of 5 × 10^6^ cells/mL, and 100 μL of this suspension was added to the upper chamber of each Transwell insert. The plates were incubated at 37°C in a 5% CO_2_ atmosphere for 4 hours. Following incubation, cells that had migrated to the lower chamber were collected. Migrated cells were quantified using a flow cytometer on a BD LSRFortessa X-29 analyzer. All experiments were performed in triplicate, and data were analyzed using GraphPad Prism 9 software. Statistical significance was determined using one-way ANOVA followed by Tukey’s post-hoc test, with p<0.05 considered significant.

### QUANTIFICATION AND STATISTICAL ANALYSIS

All experiments were performed at least two independent repeats unless otherwise indicated, and representative results are shown in the figures. Unpaired, two-tailed Student’s t-test was used to compare the two groups (treatments vs. control). One-way ANOVA models with post hoc comparisons were conducted using Tukey’s or Dunnett’s adjustments to compare continuous outcomes across multiple experimental groups. The statistical details including the statistical tests used, exact value of n, are all specified in the figure legends. For all analyses, data are reported as the mean ± SD. Statistical analysis was performed using GraphPad Prism (version 9.04). Statistical significance was defined as p <= 0.05.

#### Tumor growth curve

Growth curves were examined using a repeated-measures model to consider the within-mouse correlation. Overall survival was illustrated with Kaplan-Meier curves, and group comparisons were made using log rank tests. All analyses were carried out using GraphPad Prism, version 9.04 (GraphPad Software).

#### Single cell-RNA sequencing

Clustering of single cells, UMAP, violin plots, and marker gene analyses were conducted using R with Seurat 4.1.3.^50^ log_2_(TPM+1) values were used for Seurat analyses and were normalized by regressing out the number of detected features and the percent of mitochondrial reads. Cells with fewer than 200 features or genes present in fewer than 3 cells were filtered out. The top differentially expressed genes in each cluster were identified with the Wilcoxon rank-sum test (min.pct = 0.25, logfc.threshold = 0.25) and used to discern cluster identities. Statistical analysis of changes in cluster proportion across conditions was performed with the Single Cell Proportion Test library.^54^

## Supplementary Material

Supplementary material

SUPPLEMENTAL INFORMATION

Supplemental information can be found online at https://doi.org/10.1016/j.celrep.2025.115893.

## Figures and Tables

**Table:** KEY RESOURCES TABLE

REAGENT or RESOURCE	SOURCE	IDENTIFIER
Antibodies		
TruStain FcX anti-mouse CD16/32) antibody	Biolegend	Cat# 101320); RRID:AB_1574975
anti-mouse CD45.2(30-F11)	Biolegend	Cat# 103147; RRID:AB_2564383
Purified Rat Anti-Mouse CD16/CD32 BD (Mouse BD Fc BlockTM) Clone 2.4G2	BD Biosciences	Cat# 553141; RRID:AB_394656
TCR-beta (H57–597)	Biolegend	Cat# 109202; RRID:AB_313425
CD4 (GK1.5)	Biolegend	Cat# 100411; RRID:AB_312696
CD8 (53–6.7)	Biolegend	Cat# 100712; RRID:AB_312751
NK1.1 (PK136)	Biolegend	Cat# 108708; RRID:AB_313395
CD11b (M1/70)	Biolegend	Cat# 101206; RRID:AB_312789
CD11c (N418)	Biolegend	Cat# 117333; RRID:AB_11204262
MHCII (M5/114.15.2)	Biolegend	Cat# 107621; RRID:AB_493726
Ly6G (1A8)	Biolegend	Cat# 127607; RRID:AB_1186104
F4/80 (BM8)	Biolegend	Cat# 123131; RRID:AB_10901171
CD206 (MMR)	Biolegend	Cat# 141720; RRID:AB_2562248
Arginase-1	Invitrogen	Cat# 17–3697-80; RRID:AB_2734834
IFN-Gamma	Biolegend	Cat# 505808; RRID:AB_315402
IL-10	Biolegend	Cat# 505028; RRID:AB_2561523
CD14	BD Biosciences	Cat# 555399;RRID: AB_398596; clone M5E2
CD15	BD Biosciences	Cat# 562371; RRID:AB_11154049; clone W6D3
CD33	BD Biosciences	Cat# 340474, RRID:AB_400518; clone P67.6
HLA-DR	BD Biosciences	Cat# 339194; RRID:AB_647443; clone L243
CD11b	BD Biosciences	Cat# 557743; RRID:AB_396849; clone ICRF44
LOX-1	Biolegend	Cat# 358610; RRID:AB_2728343; clone 15C4
Arg-1	E-Biosciences	Cat# 17–3697-82; RRID:AB_2734835
SG7	Cochran lab	NA
Dead-Fc anti-VISTA	Cochran lab	NA
Anti-VISTA	Hummingbird Biosciences Inc, Singapore	#HMBD-002
Biological samples		
PBMCs from HNC	Stanford University Medical Center	N/A
Chemicals, peptides, and recombinant proteins		
Ammonium chloride potassium (ACK)	Gibco	#A1–49201
FBS	Sigma	#F0926
FCS	Sigma	#12133C
Antibiotics	Gibco	#15240062
Tumor dissociation kit, mouse	Miltenyi	#130–096-730
Zombie NIR Fixable viability kit	Biolegend	#423106
FIX & PERM Cell Permeabilization kit	ThermoFisher	#GAS004
Fixation-Permeabilization kit	eBioscience	#88–8824-00
RPMI-1640	ThermoFisher	#11875093
0.1 mg/ml of DNase I	Sigma	#11284932001
1 mg/ml collagenase	Sigma	#1108866001
40 μm cell strainers	Corning	#352340
Red Blood Cell Lysis Solution	Miltenyi	#130–094-183
Human FcR Blocking Reagent	MACS™; Miltenyi Biotec	#130–059-901
HBSS	Gibco	#14025092
Deposited data		
scRNA-seq data	GSE295914	This study
Code	https://doi.org/10.5281/zenodo.15192114	This study
Experimental models: Cell lines		
MOC1	Laboratory of Ravindra Uppaluri at Washington University in Saint Louis.	N/A
MOC2	Laboratory of Ravindra Uppaluri at Washington University in Saint Louis.	N/A
P029	University of Colorado by XJ Wang	N/A
B16L6	AcceGen	(#ABC-TC0060)
4T1	ATCC	(#CRL-2539)
Experimental models: Organisms/strains		
C57BL/6	Charles River Laboratories	027 (Strain code)
Balb/c	Charles River Laboratories	028 (Strain code)
*VSIR*−/− mice	Mutant Mouse Regional Resource Centers (www.mmrrc.org)	Stock no. 031655-UCD; B6;129S5-Vsirtm1Lex/Mmucd
Software and algorithms		
Biorender	Biorender	N/A
FlowJo 10.8.1	Becton Dickinson & Company	RRID: SCR_008520
FlowJo software (Tree Star)		N/A
R package Seurat v4.0.1		N/A
Cell Ranger 7.0.1.45 (10X Genomics)		N/A
Harmony		N/A
GraphPad Prism, version 9.04	GraphPad Software Inc	RRID: SCR_002798
Cytobank Stanford	http://cytobank.org/	N/A

## Highlights

- Radiotherapy elevates VISTA expression on myeloid cells in tumors and blood
- VISTA deletion enhances myeloid repolarization and T cell activation
- VISTA blockade improves radiotherapy response in multiple preclinical models
- Interferon signaling and antigen presentation increase with VISTA loss
