# Supplementary material for: VISTA immune checkpoint blunts radiotherapy-induced antitumor immune response
Source: Cell Rep. Author manuscript; Available in PMC 2026 Jan 25. (PMC12832042; doi:10.1016/j.celrep.2025.115893)
Supplement: Supplementary material [file NIHMS2114901-supplement-Supplementary_material.pdf]

Cell Reports, Volume *44*

Supplemental information

VISTA immune checkpoint blunts radiotherapy-induced antitumor immune response

Dhanya K. Nambiar, Sainiteesh Maddineni, Jimpi Langthasa, Hongbin Cao, Vignesh Viswanathan, Junyan Liu, Md Tauhidul Islam, Nishant Mehta, Jessica Frank, Alexander Real, Tia Cheunkarndee, Eyiwunmi Eghonghon Laseinde, Bhushan Dharmadhikari, Dipti Thakkar, Jerome D. Boyd-Kirkup, Andrey Finegersh, Vasu Divi, John B. Sunwoo, John Aleman, Xiao-Jing Wang, Christina Kong, Lei Xing, Jennifer R. Cochran, and Quynh-Thu Le

Human HNSCC Sample Flow cytometry Gating

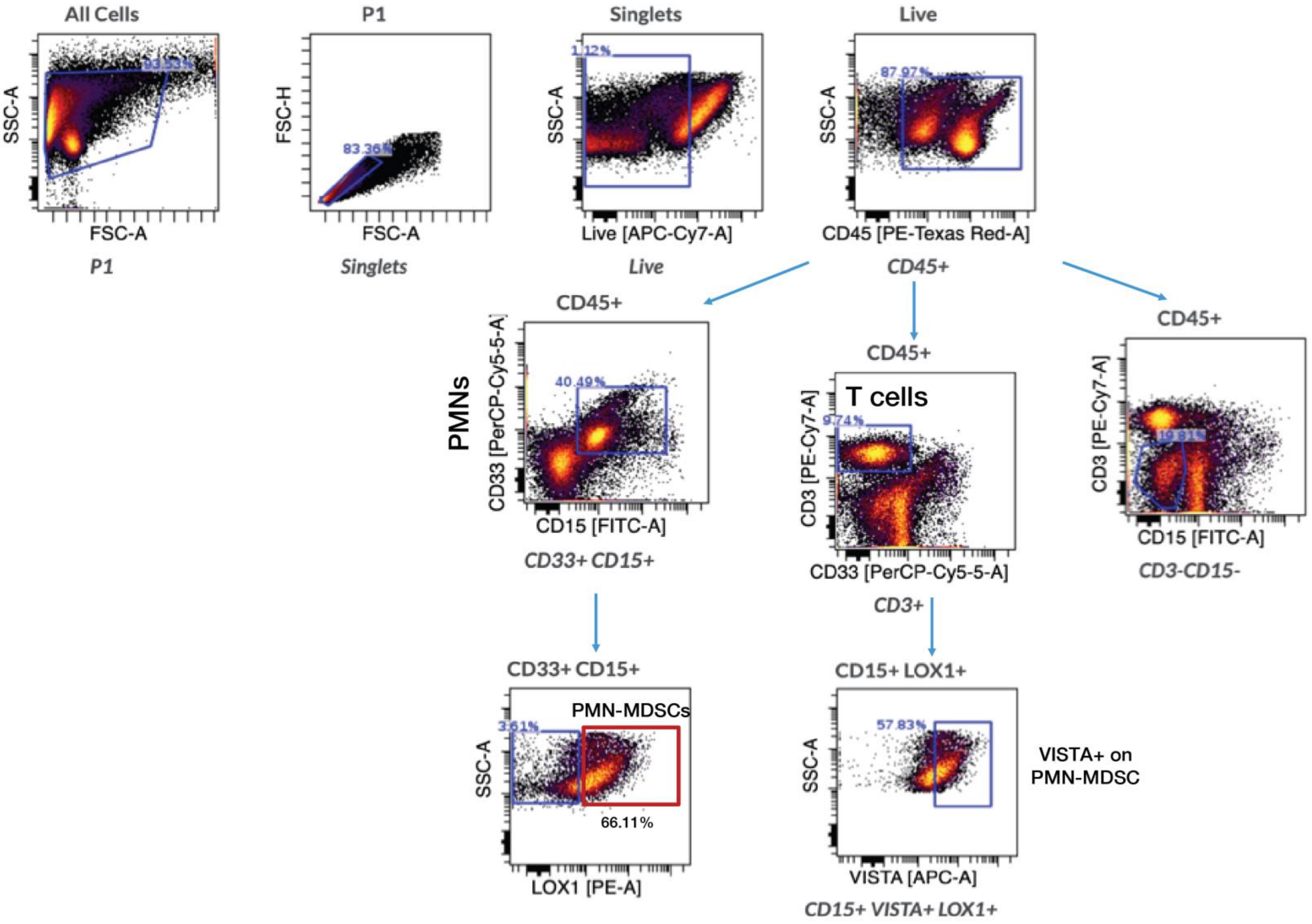

Figure S1: Gating strategy for flow cytometric analyses of VISTA expression on major myeloid and lymphoid populations in the dissociated human primary HNSCC samples (related to Figure 1).

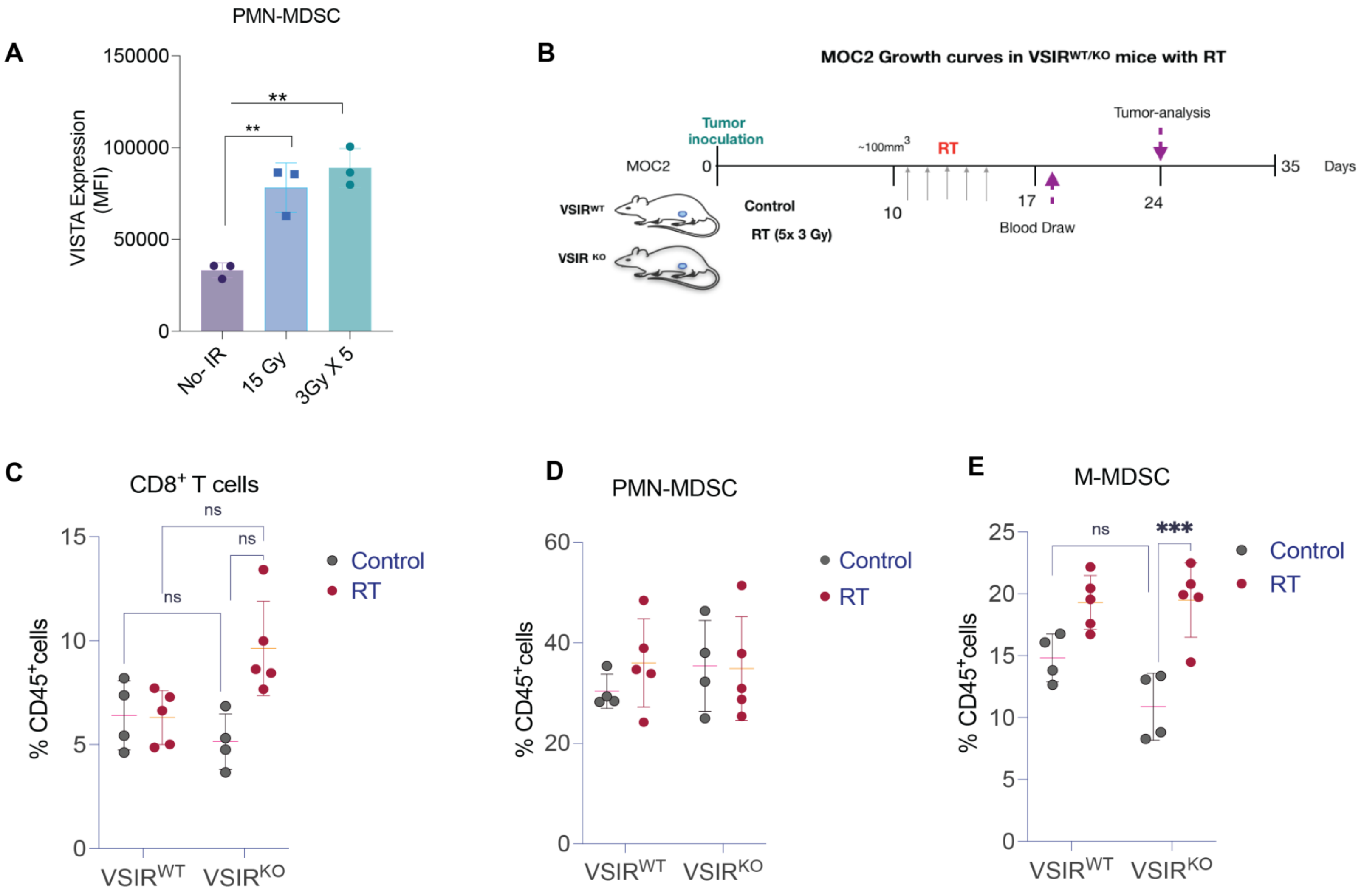
A
PMN-MDSC
VISTA Expression (MFI)
150000
100000
50000
0
**
**
No- IR
15 Gy
3Gy X 5
B
MOC2 Growth curves in VSIRWT/KO mice with RT
Tumor inoculation
Tumor-analysis
~100mm3
RT
MOC2
0
10
17
24
35
Days
VSIRWT
VSIR KO
Control
RT (5x 3 Gy)
Blood Draw
C
CD8+ T cells
% CD45+cells
15
10
5
0
ns
ns
ns
Control
RT
VSIRWT
VSIRKO
D
PMN-MDSC
% CD45+cells
60
40
20
0
Control
RT
VSIRWT
VSIRKO
E
M-MDSC
% CD45+cells
25
20
15
10
5
0
ns
***
Control
RT
VSIRWT
VSIRKO

Figure S2: Tumors grown in *VSIR*^{KO} mice show better response to RT than those grown in *VSIR*^{WT} mice (related to Figure 3). **A)** Quantification of VISTA expression (median MFI) in MOC2 tumor infiltrated PMN-MDSCs after 2 weeks post RT treatment regimens 3Gyx5 or a single dose of 15 Gy in C57/BL6 VISTA^{WT}mice (n=3/group) **B)** Schema showing the treatment and the different time points of blood and tumor analyses of MOC2 tumors implanted in *VSIR*^{WT} and *VSIR*^{KO} mice. **C-E**) Quantification of CD8^{+} T cells **(C)**, PMN-MDSCs **(D)**, and M-MDSCs **(E)** as the percent of CD45^{+} cells by flow cytometry analysis of the tumors in A (n= 4-5 mice).

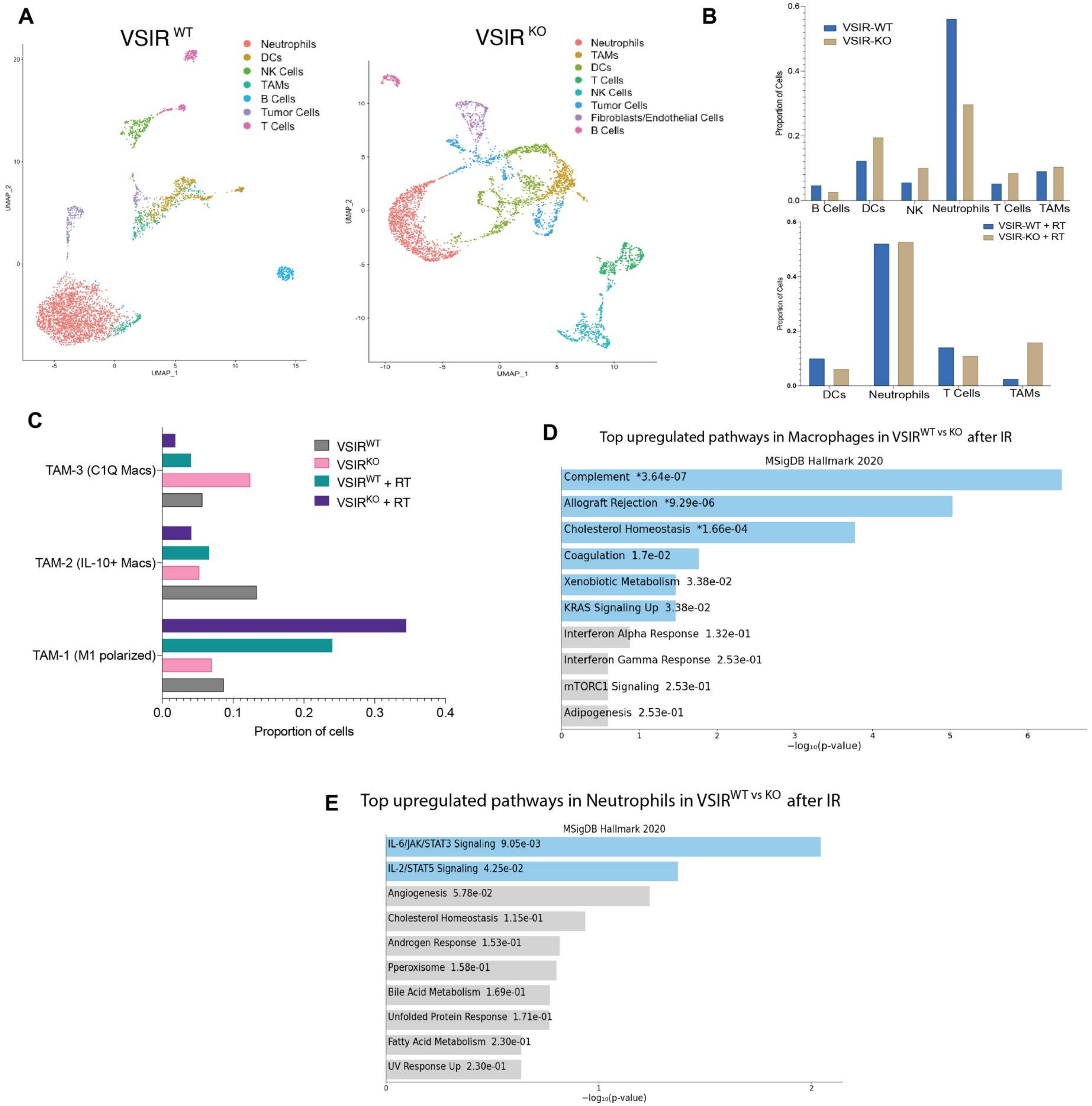
A
VSIR WT
Neutrophils
DCs
NK Cells
TAMs
B Cells
Tumor Cells
T Cells
UMAP_1
UMAP_2
VSIR KO
Neutrophils
TAMs
DCs
T Cells
NK Cells
Tumor Cells
Fibroblasts/Endothelial Cells
B Cells
B
VSIR-WT
VSIR-KO
Proportion of Cells
B Cells
DCs
NK
Neutrophils
T Cells
TAMs
VSIR-WT + RT
VSIR-KO + RT
DCs
Neutrophils
T Cells
TAMs
C
TAM-3 (C1Q Macs)
TAM-2 (IL-10+ Macs)
TAM-1 (M1 polarized)
VSIR WT
VSIR KO
VSIR WT + RT
VSIR KO + RT
Proportion of cells
D
Top upregulated pathways in Macrophages in VSIR WT vs KO after IR
MSigDB Hallmark 2020
Complement *3.64e-07
Allograft Rejection *9.29e-06
Cholesterol Homeostasis *1.66e-04
Coagulation 1.7e-02
Xenobiotic Metabolism 3.38e-02
KRAS Signaling Up 3.38e-02
Interferon Alpha Response 1.32e-01
Interferon Gamma Response 2.53e-01
mTORC1 Signaling 2.53e-01
Adipogenesis 2.53e-01
−log10(p-value)
E
Top upregulated pathways in Neutrophils in VSIR WT vs KO after IR
MSigDB Hallmark 2020
IL-6/JAK/STAT3 Signaling 9.05e-03
IL-2/STAT5 Signaling 4.25e-02
Angiogenesis 5.78e-02
Cholesterol Homeostasis 1.15e-01
Androgen Response 1.53e-01
Pperoxisome 1.58e-01
Bile Acid Metabolism 1.69e-01
Unfolded Protein Response 1.71e-01
Fatty Acid Metabolism 2.30e-01
UV Response Up 2.30e-01
−log10(p-value)

Figure S3: Single-cell sequencing analysis of MOC2 tumors implanted in *VSIR*^{WT} and *VSIR*^{KO} mice (related to Figure 4). A) Uniform manifold approximation and projection (UMAP) of cell clusters arising from an integrated dataset of tumor samples from *VSIR*^{WT} and *VSIR*^{KO} mice. **B)** Bar plots showing the changes in proportion of cells from the single cell RNA seq analyses after integrated clustering of samples. **C)** Proportion of different TAM clusters in 4 different groups of samples analyzed. Dot plot of expression of top differentially expressed genes per cluster. **D)** Gene enrichment analysis using MSigDB gene sets depicting top-up-regulated pathways in tumor macrophages isolated from MOC2 tumors grown in *VSIR*^{WT} mice compared to those in the *VSIR*^{KO} mice. **E)** Gene enrichment analysis using MSigDB gene sets depicting top-up-regulated pathways within the neutrophil cluster in the TME of the *VSIR*^{WT}-RT compared to the *VSIR*^{KO}-RT group.

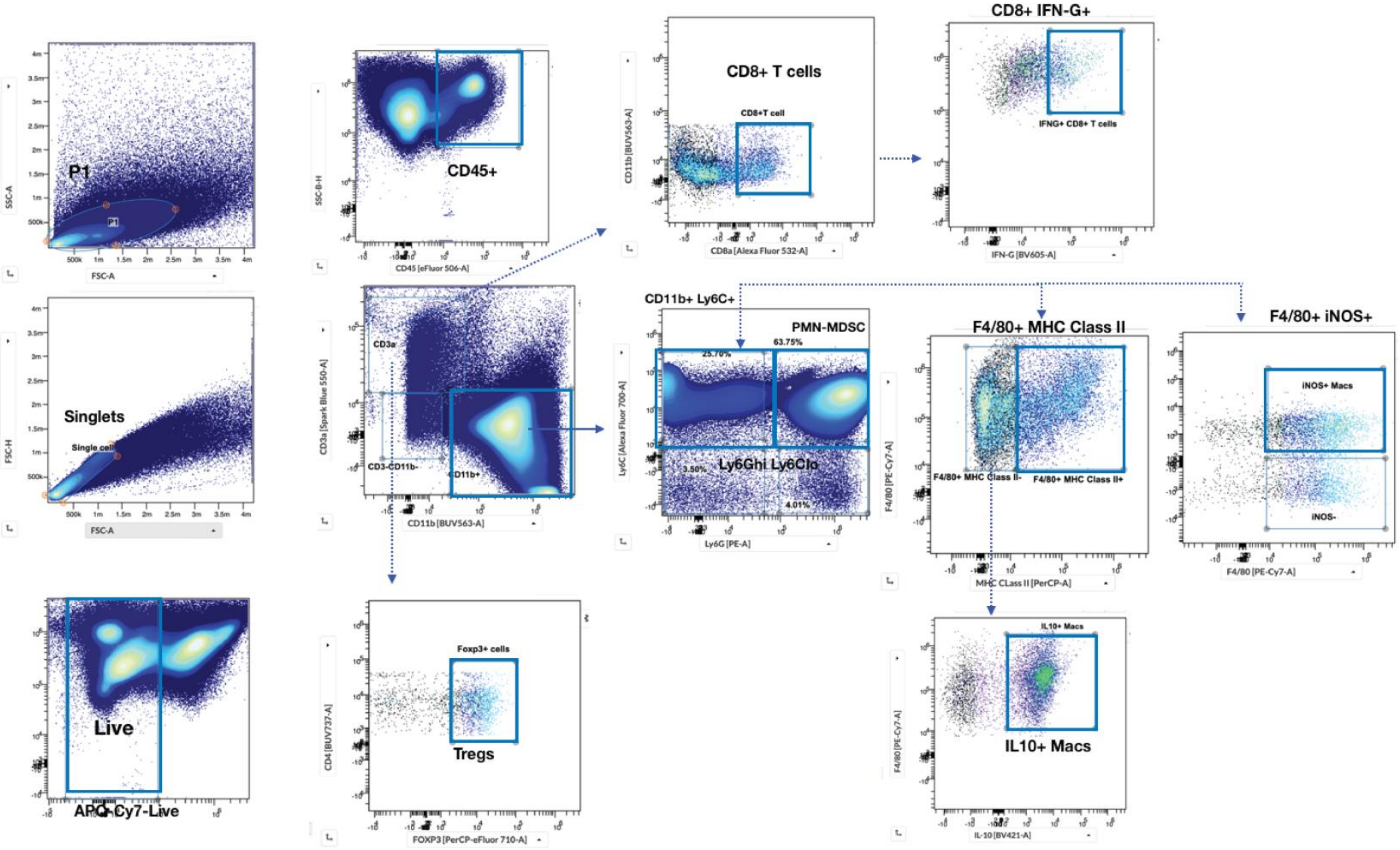

P1
Singlets
Single cell
Live
APC-Cy7-Live
CD45+
CD3a
CD3-CD11b-
CD11b+
Tregs
Foxp3+ cells
CD8+ T cells
CD8+T cell
CD8+ IFN-G+
IFNG+ CD8+ T cells
CD11b+ Ly6C+
PMN-MDSC
25.70%
63.75%
3.50%
4.01%
Ly6Ghi Ly6Clo
F4/80+ MHC Class II
F4/80+ MHC Class II-
F4/80+ MHC Class II+
F4/80+ iNOS+
iNOS+ Macs
iNOS-
IL10+ Macs
SSC-A
FSC-A
FSC-H
SSC-B-H
CD45 [eFluor 506-A]
CD3a [Spark Blue 550-A]
CD11b [BUV563-A]
CD4 [BUV737-A]
FOXP3 [PerCP-eFluor 710-A]
CD8a [Alexa Fluor 532-A]
IFN-G [BV605-A]
Ly6C [Alexa Fluor 700-A]
Ly6G [PE-A]
F4/80 [PE-Cy7-A]
MHC CLass II [PerCP-A]
IL-10 [BV421-A]

Figure S4: Gating strategy for flow cytometric analyses of different immune cell populations in the MOC2 tumors (related to Figure 7).

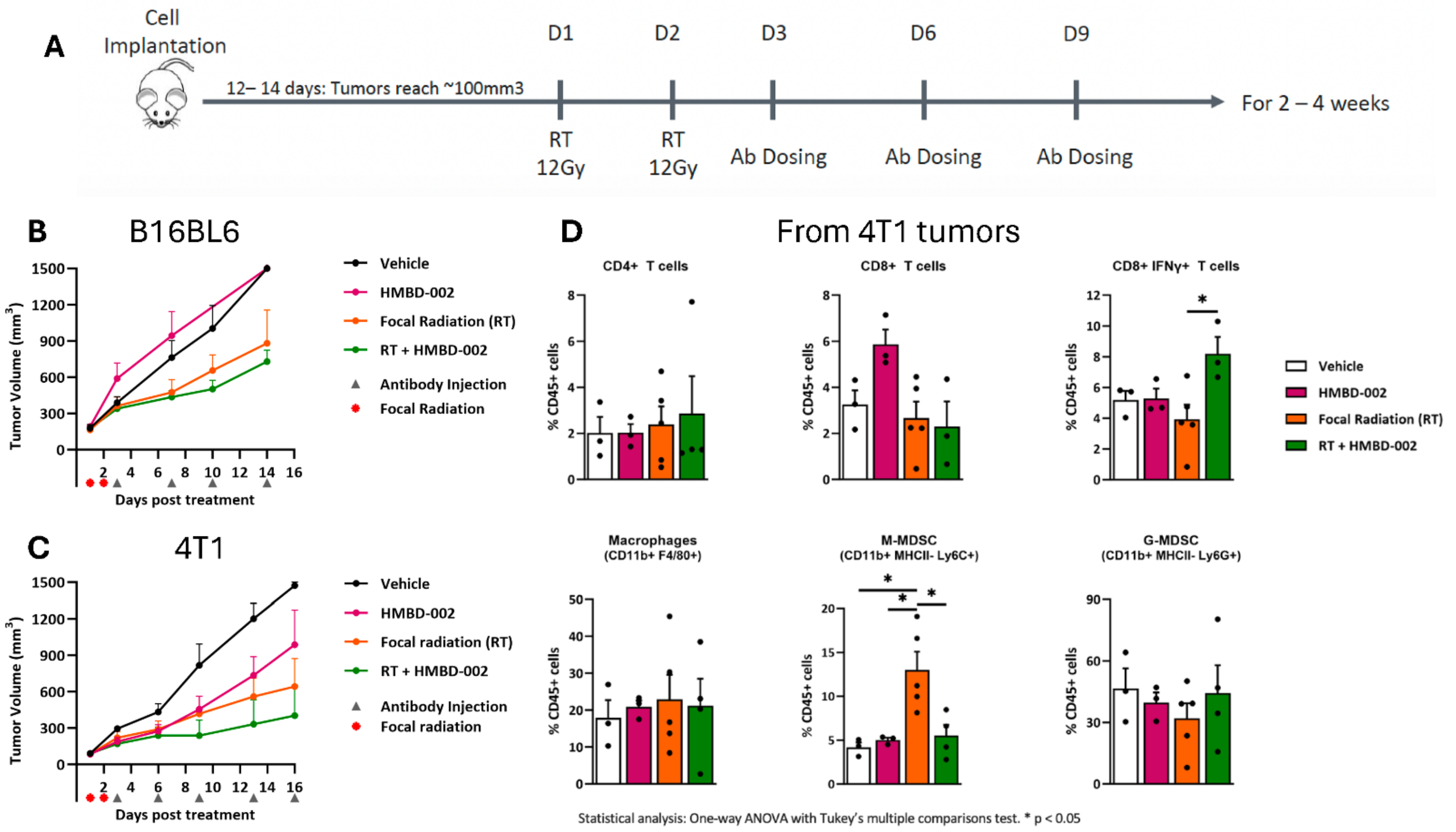
A
Cell Implantation
12– 14 days: Tumors reach ~100mm3
D1
D2
D3
D6
D9
RT 12Gy
RT 12Gy
Ab Dosing
Ab Dosing
Ab Dosing
For 2 – 4 weeks
B
B16BL6
Tumor Volume (mm3)
Days post treatment
Vehicle
HMBD-002
Focal Radiation (RT)
RT + HMBD-002
Antibody Injection
Focal Radiation
C
4T1
Tumor Volume (mm3)
Days post treatment
Vehicle
HMBD-002
Focal radiation (RT)
RT + HMBD-002
Antibody Injection
Focal radiation
D
From 4T1 tumors
CD4+ T cells
CD8+ T cells
CD8+ IFNγ+ T cells
Macrophages (CD11b+ F4/80+)
M-MDSC (CD11b+ MHCII- Ly6C+)
G-MDSC (CD11b+ MHCII- Ly6G+)
% CD45+ cells
Vehicle
HMBD-002
Focal Radiation (RT)
RT + HMBD-002
Statistical analysis: One-way ANOVA with Tukey's multiple comparisons test. * p < 0.05

Figure S5: Anti-VISTA antibody enhances response to RT in B16BL6 melanoma and 4T1 breast cancer model (related to Figure 5). A) Schema showing the timing of tumor implantation and the different treatments. **B)** Tumor growth curves showing the response of B16L6 melanomas in C57/BL6 mice treated with vehicle, anti-VISTA antibody (HMBD-002), RT alone (12Gyx2), and RT + anti-VISTA antibody (HMBD-002) (N = 7 mice/ group). **C)** Tumor growth curves showing the response of 4T1 breast cancer in Balb/c mice treated with vehicle, anti-VISTA antibody (HMBD), RT alone (12Gyx2), and RT + anti-VISTA antibody (HMBD-002) (N = 4 mice/ group). D) level of different intratumoral immune cells as a percent of $CD45^{+}$ cells isolated from a different set of 4T1 tumors receiving the same types of treatment as in C (N = 3-5 mice).

Table S1: Distribution of patient and tumor characteristics (related to Star Methods and Figure 1).

Patient	Gender	Age	Race	Ethnicity	Primary Tumor Site	Analyzed specimens	HPV status	Pathological Staging	Recurrence
1	Male	55	Other	Hispanic/Latino	Oropharynx (Tonsil)	Recurrent node	Pos	RecT0N1M0	N
2	Female	55	White	Non-Hispanic/Latino	Oropharynx (Tonsil)	Primary tumor	Pos	T1N1M0	N
3	Female	54	Asian	Non-Hispanic/Latino	Oropharynx (Tonsil)	Primary tumor	Pos	T2N1M0	Y
4	Male	66	White	Non-Hispanic/Latino	Oral Cavity (Tongue)	Primary tumor	N/A	T2N3bM0	Y
5	Male	68	White	Non-Hispanic/Latino	Oropharynx (Base of Tongue)	Primary tumor	Pos	T2N1M0	N

Table S2: Distribution of patient, tumor, and treatment characteristics (related to Star Methods and Figure 2).

Patient	Gender	Age	Race	Ethnicity	Primary Tumor Site	HPV status	Staging Clinical or pathological	Treatment	Recurrence
1	Male	62	White	Non-Hispanic/Latino	Oropharynx (Glossotonsillar Sulcus)	Pos	cT1N2M0	ChemoRT	N
2	Male	79	White	Non-Hispanic/Latino	Oropharynx (Base of Tongue)	Pos	cT1N2M0	ChemoRT	Y
3	Male	81	White	Non-Hispanic/Latino	Oropharynx (Soft Palate)	Neg	cT2N0M0	RT	N
4	Male	67	White	Non-Hispanic/Latino	Oropharynx (Tonsil)	Pos	cT2N1M0	ChemoRT	N
5	Male	73	White	Non-Hispanic/Latino	Oropharynx (Glossotonsillar Sulcus)	Pos	cT2N2M0	ChemoRT	N
6	Male	69	White	Non-Hispanic/Latino	Larynx (Supraglottis)	N/A	cT3N0M0	ChemoRT	N
7	Male	76	American Indian or Alaska Native	Non-Hispanic/Latino	Oropharynx (Tonsil)	Pos	cT4N1M0	RT + Immunotherapy	N
8	Male	81	White	Non-Hispanic/Latino	Oral Cavity (Mandible)	N/A	pT4aN0M0	Surgery + ChemoRT	N
9	Male	82	White	Non-Hispanic/Latino	Cutaneous SCC (Ear)	Neg	pT1N3bM0	Surgery + RT	N
10	Male	89	White	Non-Hispanic/Latino	Oral Cavity (Mandibular Alveolar Ridge)	N/A	cT4aN2bM0	RT (palliative)	N
11	Male	84	White	Non-Hispanic/Latino	Oropharynx (Base of Tongue)	Pos	cT2N1M0	RT	N
12	Female	74	White	Non-Hispanic/Latino	Oropharynx (Tonsillar Fossa)	Pos	cT1N1M0	ChemoRT	N
13	Female	74	White	Non-Hispanic/Latino	Oral Cavity (Tongue)	Neg	pT1N2bM0	Surgery + RT	N
14	Male	77	White	Non-Hispanic/Latino	Oropharynx (Base of Tongue)	Pos	cT2N1M0	RT	N
15	Male	78	Asian	Non-Hispanic/Latino	Oropharynx (Tonsil)	Pos	cT1N1M0	ChemoRT	N
16	Male	65	White	Non-Hispanic/Latino	Larynx (Supraglottis)	Neg	pT4aN3bM0	Surgery + ChemoRT	Y
